# Harnessing blue light photobiomodulation for cancer therapy: Evidence from a systematic review

**DOI:** 10.1111/php.70025

**Published:** 2025-08-28

**Authors:** Bárbara Evelyn Santos de Lima, Rebeca Barros Nascimento, Ana Paula Mariano Santos Ginez, Maria Stella Moreira, Rebeca Boltes Cecatto, Rodrigo Labat Marcos, Maria Fernanda Setúbal Destro Rodrigues

**Affiliations:** ^1^ Medicine‐Biophotonics PostGraduate Program Universidade Nove de Julho/UNINOVE Sao Paulo Brazil; ^2^ Department of Stomatology, School of Dentistry University of São Paulo Sao Paulo Brazil; ^3^ Department of Oral Medicine A.C. Camargo Cancer Center Sao Paulo Brazil

**Keywords:** animal models, blue light, cancer, in vitro studies, photobiomodulation, systematic review

## Abstract

Cancer is a chronic disease responsible for millions of deaths annually. Its multifaceted profile, with diverse types and anatomical locations, complicates treatment, often limited to surgery, radiotherapy, and chemotherapy. These treatments are frequently associated with increased tumor aggressiveness and recurrence, highlighting the urgent need for new, less invasive therapies. Recent studies suggest that blue light (BL; 450–470 nm) may offer anti‐tumor and pro‐apoptotic effects, making it a promising alternative for cancer treatment. However, its cellular and molecular mechanisms remain unclear. This qualitative systematic review, conducted in accordance with PRISMA guidelines, analyzed 37 in vitro and in vivo studies published between 2002 and 2024, retrieved from databases including MEDLINE/PubMed, EMBASE, and LILACS, with a focus on the effects of photobiomodulation (PBM) with blue light (450–470 nm) in pre‐clinical cancer models. BL demonstrated anti‐tumor potential by reducing cell viability, proliferation, migration, and invasion, as well as increasing ROS production and inducing apoptosis. In animal models, BL also inhibited tumor growth, metastasis, and improved survival. Despite the encouraging findings, considerable methodological heterogeneity and insufficient reporting of dosimetric parameters compromise the reproducibility and comparability of results across studies. These findings underscore the therapeutic potential of BL in oncology and highlight the need for standardized protocols to support clinical translation.

## Graphical abstract

Conventional treatments for various types of cancer are often associated with tumor recurrence and aggressiveness. Photobiomodulation with blue light (BL; 450–470 nm) emerges as a promising alternative with antitumor effects. This systematic review, with 37 in vitro and in vivo studies from 2002 to 2024, revealed that BL reduces cell viability, proliferation, migration, apoptosis, and invasion. In addition, BL induces ROS production and inhibits tumor growth and metastasis. Despite its therapeutic potential, standardization of protocols is needed for future clinical application.

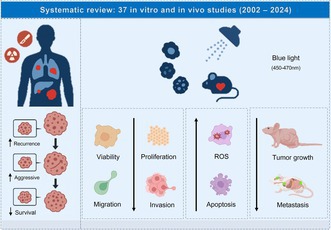

**Abbreviations**
AHRQ: Agency for Healthcare Research and Quality
AKT2: AKT serine/threonine kinase 2
AML1: acute myeloid leukemia 1
ASK1: apoptosis signal‐regulating kinase 1
ATP: adenosine triphosphate
BL: blue light
CADTH: Canadian Agency for Drugs and Technologies in Health
CAPES: Coordenação de Aperfeiçoamento de Pessoal de Nível Superior
CCK: cell counting kit
CCL7: chemokine (C‐C motif) ligand 7
CD163: cluster of differentiation 163
CDK2: cyclin‐dependent kinase 2
CDK4: cyclin‐dependent kinase 4
CEP: Código de Endereçamento Postal (Brazilian postal code)
DCFDA: 2′,7′‐dichlorofluorescin diacetate
DDIT3: DNA damage inducible transcript 3 (also known as CHOP)
DHE: dihydroethidium
DNA: deoxyribonucleic acid
ECONLIT: economics literature database
EMBASE: excerpta medica database
ERK: extracellular signal‐regulated kinase
ERK1/2: extracellular signal‐regulated kinases 1 and 2
FAPESP: Fundação de Amparo à Pesquisa do Estado de São Paulo
GLOBOCAN: global cancer observatory
HEN: health evidence network
HPSE: heparanase
HSPA5: heat shock protein family A (Hsp70) member 5
ICTRP: International Clinical Trials Registry Platform
INAHTA: International Network of Agencies for Health Technology Assessment
JNK: c‐Jun N‐terminal Kinase
LDH: lactate dehydrogenase
LILACS: Latin American and Caribbean Health Sciences Literature
LIVIVO: life sciences literature database
MAPK: mitogen‐activated protein kinase
MAUDE: manufacturer and user facility device experience
MEDLINE: medical literature analysis and retrieval system online
MEK: mitogen‐activated protein kinase kinase
MMP: matrix Metalloproteinases (MMP2, MMP9, MMP10)
MTS: 3‐(4,5‐dimethylthiazol‐2‐yl)‐5‐(3‐carboxymethoxyphenyl)‐2‐(4‐sulfophenyl)‐2H‐tetrazolium
MTT: 3‐(4,5‐dimethylthiazol‐2‐yl)‐2,5‐diphenyltetrazolium bromide
MYC: MYC proto‐oncogene
NARIC: National Rehabilitation Information Center
NIR: near infrared
NOX5: NADPH oxidase 5
PARP: poly (ADP‐ribose) polymerase
PBM: photobiomodulation
PCNA: proliferating cell nuclear antigen
PDT: photodynamic therapy
PI: propidium iodide
PICOS: population, intervention, comparator, outcomes, study design
PRISMA: preferred reporting items for systematic reviews and meta‐analyses
PROSPERO: International Prospective Register of Systematic Reviews
QUIN: quality assessment tool for in vitro studies
REHABDATA: rehabilitation research database
RIPSA: Rede Interagencial de Informações para a Saúde (Brazilian Interagency Health Information Network)
ROS: reactive oxygen species
SCOPUS: Elsevier Abstract and Citation Database
SET: SET Nuclear Oncogene
SRC: SRC proto‐oncogene, non‐receptor tyrosine kinase
SYK: spleen tyrosine kinase
SYRCLE: systematic review centre for laboratory animal experimentation
TUNEL: terminal deoxynucleotidyl transferase dUTP nick end labeling
UNINOVE: Universidade Nove de Julho
VEGF: vascular endothelial growth factor
WB: Western Blot
WHO: World Health Organization
WST‐1: water‐soluble tetrazolium salt‐1
XBP1: X‐box binding protein 1

## INTRODUCTION

Cancer is a worldwide public health and economic problem that affects the life expectancy and quality of life of millions of people annually. This disease is among the main causes of death in developed countries, representing the first or second cause of premature death before the age of 70.^1^ According to the Global Cancer Observatory (GLOBOCAN), there were 20 million new cases of cancer worldwide in 2022, and it is estimated that one in five people will have cancer at some point in their life.^1^ Therapeutic alternatives have been explored to assist conventional cancer treatments, especially for patients with advanced stages of the disease. Photobiomodulation (PBM) with blue light (BL) has shown promising results in some in vitro and in vivo models of different types of cancer.^2^, ^3^, ^4^ However, due to the diversity of dosimetric parameters, the use of different in vivo models, and methodological strategies in studies with light in cancer, the effective parameters of blue PBM are not yet well established, as well as its mechanisms of action in tumor cells that lead to pro or antitumor effects.

This study performed a systematic review of the literature on the effect of PBM with BL in in vitro and in vivo models of different types of cancer, aiming to contribute to the understanding of the biological effects and mechanism of action of this therapy, as well as to discuss the most promising dosimetric parameters with translational potential. This approach not only reduces the possibility of bias in the interpretation of results, but also allows the identification of gaps in knowledge, guiding future investigations in oncology therapy.

## MATERIALS AND METHODS

This systematic literature review was performed in 2024 covering the period from 2002 to 2024 and following the Preferred Reporting Items for Systematic Reviews and Meta‐Analysis (PRISMA Statement) guidelines.^5^ This protocol was registered on the PROSPERO website before data extraction (registration no. CRD42023446218).

### Search strategy

Study selection was guided by the PICOS strategy: Population: in vitro and in vivo studies; Intervention: blue light; Comparator: any comparator; Outcomes: in vitro (reduction of cell viability, cell death, decrease of metastatic and invasive profiles) – and in vivo (increase of survival, tumor decrease, and decrease of metastatic and invasive profiles; Study type: experimental studies).

In November of 2024, the systematic review was performed by two independent reviewers (BESL and MFSDR), and a third one in cases of disagreement, searching by studies published in English, Spanish, French, Portuguese, or Italian. Different databases were consulted to identify relevant studies: MEDLINE/PubMed, EMBASE, LILACS, Open Gray, Proquest, CADTH Database, Interinstitutional Network of Health Information (RIPSA), International Network of Agencies of Health Technology Assessment (INAHTA) Health Evidence Network (HEN WHO), Agency for Healthcare Research and Technology Assessments (AHRQ), MAUDE – Manufacturer and User Facility Device Experience, International Clinical Trials Registry Platform (ICTRP), Physiotherapy Evidence Database (PEDro), National Rehabilitation Information Center (NARIC), REHABDATA database, ECONLIT, EconPapers, Cochrane database, Livivo, and Scopus. The resource Ryyan (https://www.rayyan.ai/) was used to help the screening and selection of studies.

### Eligibility criteria

The inclusion criteria of studies were all studies that evaluated the effect of blue light PBM in in vitro or in vivo models, with any comparison. Systematic reviews and meta‐analyses that aim to investigate the same purpose of this systematic review were also included. Exclusion criteria were clinical studies; studies developed in silico and studies without a comparator.

### Data extraction

A standard spreadsheet of data extracted included: author and year of publication; type of study; in vitro and/or in vivo studies; outcome assessment methods; control and treatment groups; irradiation parameters; results/outcomes.

### Analysis of risk of bias (RoB)

The risk of bias was evaluated using the resource SYRCLE for in vivo studies and using the QUIN for in vitro studies.^6^, ^7^

## RESULTS

### Search results

**FIGURE 1:**
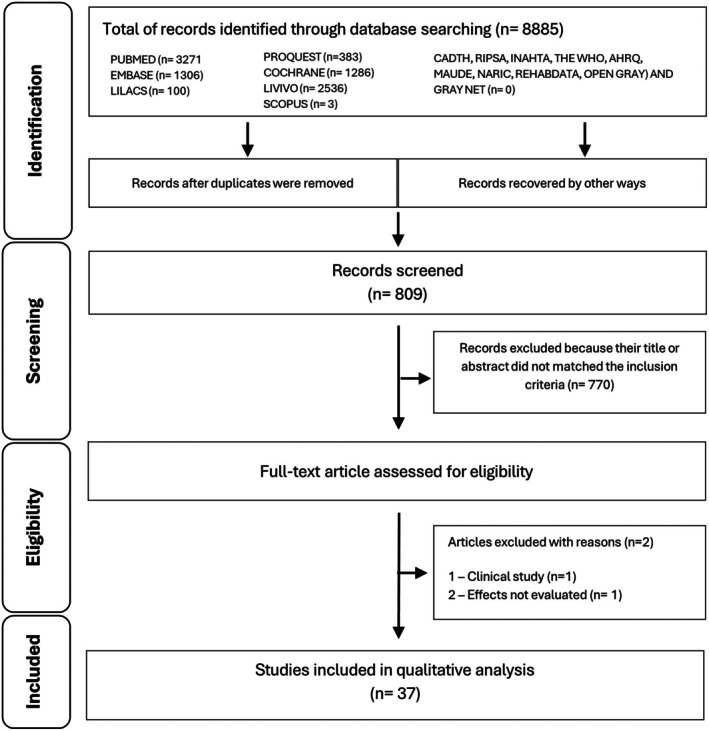
Flow diagram of the literature search and selection criteria adapted from PRISMA (Moher et al., 2010).

From the survey in different databases and after eliminating duplicates, 37 articles were included and analyzed in this systematic review (Figure 1). The search strategies retrieved 8885 references: 3271 on PUBMED, 1306 on EMBASE, 1286 on Cochrane Library, 100 on LILACS, 383 on PROQUEST, 2536 on LIVIVO, 3 on SCOPUS, and none on CADTH, RIPSA, INAHTA, THE WHO, AHRQ, MAUDE, NARIC, REHABDATA, and OPEN GRAY. One additional record was identified through a hand search. A total of 8077 duplicate references were removed, and titles and abstracts screened 808 references. Of these, 39 studies were analyzed in full text, and two of them were excluded (Wang 2024 and Rosow 2024) for the following reasons: no evaluation of the effect of BL on tumor cells and clinical study, respectively.^8^, ^9^ Thus, we included 37 studies in this systematic review. The flowchart of the search and screening of the studies for the present review is shown in Figure 1.

**FIGURE 2:**
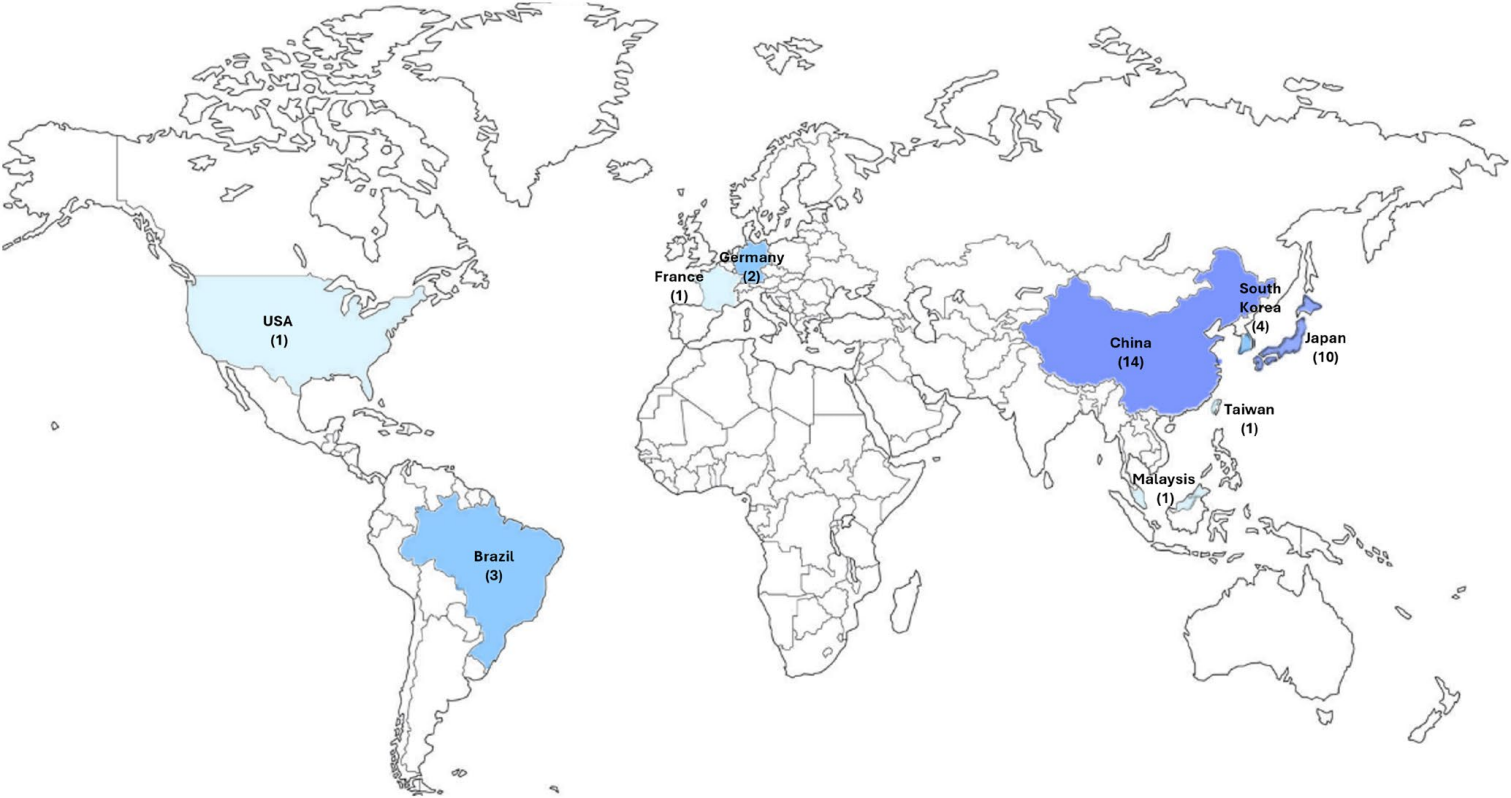
Graphical presentation showing the countries of origin of the publications included in this review.

Thirty‐six articles developed in vitro experiments, 26 of which were based only on in vitro methodologies, 10 articles used in vitro and in vivo methodologies, and one article performed only in vivo methodology (Table S1). China (*n* = 14) and Japan (*n* = 10) presented the largest number of publications, followed by Korea (*n* = 4) and Brazil (*n* = 3) (Table S1 and Figure 2).

### In vitro

Melanoma was the most studied cancer type in in vitro studies,^10^, ^11^, ^12^, ^13^, ^14^, ^15^, ^16^ followed by colorectal cancer,^4^, ^17^, ^18^, ^19^, ^20^, ^21^ breast cancer,^22^, ^23^, ^24^, ^25^ oral cancer,^26^, ^27^, ^28^, ^29^ bladder and urothelial carcinoma,^30^, ^31^, ^32^ hepatic cancer,^3^, ^4^, ^33^ pancreas,^4^, ^34^ leukemia,^6^, ^35^ lymphoma,^4^, ^36^ lung,^4^, ^37^ osteosarcoma,^38^, ^39^ skin,^40^ synovial sarcoma,^2^ and Papillary thyroid carcinoma.^41^

All studies used conventional 2D cell culture to assess the in vitro effects of PBM with BL on various cellular processes and molecules (Table 1). Cell viability analysis was performed in most studies using conventional methods such as MTT, MTS, CCK‐8, and trypan blue staining, with few studies using the WST‐1 assay.^6^, ^10^, ^22^, ^25^, ^36^ Flow cytometry techniques, clonogenic assay, and/or the use of specific markers, such as CCK‐8, BrdU, and EdU, were the most used markers to evaluate cell proliferation.^2^, ^4^, ^10^, ^16^, ^18^, ^19^, ^21^, ^27^, ^28^, ^29^, ^30^, ^33^, ^34^, ^35^, ^37^, ^39^, ^41^ The expression of proteins associated with apoptosis was investigated in most of the studies through the evaluation of Annexin V, PI, TUNEL, and Hoechst 33342.^2^, ^3^, ^15^, ^16^, ^18^, ^19^, ^21^, ^28^, ^29^, ^30^, ^31^, ^32^, ^33^, ^34^, ^35^, ^36^, ^38^, ^39^ The protein and gene expression of Caspase 3, 9, and 8, Beclin 1, PARP, MAPK, among others, were evaluated by WB, qPCR, and Microarray in 19 studies.^2^, ^3^, ^4^, ^14^, ^18^, ^19^, ^20^, ^27^, ^29^, ^30^, ^33^, ^34^, ^35^, ^36^, ^37^, ^38^, ^39^, ^41^ Moreover, three studies have evaluated the total transcriptome after PBM with BL.^28^, ^29^, ^38^ The production of reactive oxygen species (ROS) was analyzed by 21 studies using methods that varied between specific dyes and probes such as DHE and DCFDA.^2^, ^3^, ^11^, ^12^, ^13^, ^14^, ^15^, ^16^, ^19^, ^26^, ^28^, ^29^, ^31^, ^32^, ^35^, ^36^, ^37^, ^39^, ^40^, ^41^

**TABLE 1:** Summary of key Characteristics of in vitro studies.

Author	Cell line	Cancer type	Study groups	Cell viability	Proliferation	Apoptosis	Protein expression	Gene expression	ROS detection	Others	Rob (QUIN)
Ohara et al.^10^	B16F10	Melanoma	Control, blue LED, red LED, green LED, and UV LED	WST‐1 assay 72 h and 120 h after irradiation	WST‐1 assay 24, 48, and 72 h after irradiation, flow cytometry (PI) after 24, 48, 72, and 96 h, BrdU and colony formation	–	–	–	–		L
Ohara et al.^6^	HL60	Leukemia	Control and blue LED	WST‐1 assay 9 and 12 days after irradiation	–	–	–	–	–	–	L
Lockwood et al.^26^	NHEK, C2507, OSC2	Squamous cells carcinoma of the gum	Control and blue LED	–	–	–	–		HFLUOR‐DA probe assay, 0, 15, 30, 45, 60, and 90 min after irradiation.	–	L
Sparsa et al.^11^	B16F10 and EJG	Melanoma	Control, blue LED, and red LED	MTT assay before, immediately after irradiation and after 24, 48, 72, and 96 h.	–	–	–	–	Lipid peroxidation assay.	–	L
Sato et al.^12^	B16F1 and B16F10	Melanoma	Control, blue LED, red LED, green LED, and yellow LED	Trypan blue exclusion after 3, 6, 12, 24, and 36 h.	Exclusion by trypan blue after 72 h	–	–	–	Flow cytometry with Hydroethidine for detection of superoxide anion.	Analysis of mitochondrial membrane potential after 6, 12, 24, and 36 h.	L
Patel et al.^27^	A431	Oral squamous cell carcinoma	Control and blue LED	MTT assay at 24, 48, and 72 h after irradiation.	Flow cytometry (PI) after 5 days of irradiation.	–	WB for ZNRF2 and NFKB after 30 min, 1, 2, and 6 h of treatment.	–	–	–	L
Matsumoto et al.^21^	HT29, HCT116 and CSC‐2FO	Colon cancer	Control, blue LED, red LED, and green LED	–	CCK‐8 assay after 1, 3, and 5 days and flow cytometry (PI) after 5 days.	Flow cytometry for annexin V and PI.	–	RT‐PCR to the genes *CASP3, CASP8, CASP9, TNFα, FAS, ERK1/2, P38, JNK, MAPK8, LC3*, and *BECN1*.	–	–	L
Oh et al.^36^	A20 and RAMOS	Lymphoma	Control and blue LED	WST‐1 assay, 1, 2, 3, and 4 h after irradiation.	–	Flow cytometry for annexin V and PI and TUNEL assay.	Wb for PARP, CASP3, cleaved CASP3, BECN2, BAX, and β‐Actin.	–	O2• assay with DHE, analyzed by flow cytometry.	Analysis of mitochondrial membrane potential by flow cytometry with Rhodamine 123, siRNA for ATG5, BECN1, and transmission electron microscopy	L
Oh et al.^20^	CT‐26 and HT‐1080	Colon cancer and fibrosarcoma	Control and blue LED	MTT assay 24 and 48 h after irradiation.	–	–	WB to MMP9, MMP2, p‐44/42, p38, NFkB, and GAPDH.	–	–	Electrophoresis and spectrometry for detection of TCTP, LASP1, Enol 1, PLS3, and GAPDH, migration and invasion assay with transwell after 0 and 6 h.	L
Yan et al.^19^	SW620 and HT29	Colon adenocarcinoma	Control, blue LED, and drug	Trypan blue exclusion assay.	EdU assay and immunohistochemistry for Ki‐67	Flow cytometry to anexin V.	WB toTWIST1, ECAD, NCAD, VIM, and GAPDH.	RT‐qPCR to *ECAD* and *VIM*	ROS formation assay with DCFH‐DA fluorescent probe.	Migration assay after 0, 24, 48, and 72 h of irradiation, immunohistochemistry for γH2A.X	L
Yoshimoto et al.^18^	HT‐29 and HCT‐116	Colon cancer	Control and blue LED	–	CCK‐8 assay after 48 h.	Flow cytometry to annexin V and PI after 24 h.	WB to OPN3 and NF023.	RT‐qPCR to *CASP3, CASP8, LC‐3, BECN1*, and *GAPDH*.	–	Fluorescence assay for autophagy analysis after 24 h, siRNA for the OPN3 gene and immunohistochemistry for OPN3.	L
Kim et al.^34^	H6c7, PaCa‐2, PANC‐1, and BxPC‐3	Pancreatic cancer	Control, blue LED, and orange LED	MTT assay 3 or 5 h after irradiation for 5 days.	BrdU assay, colony formation, and cell cycle by flow cytometry (PI).	Flow cytometry to anexin V.	WB to Poli‐polimerase, TP53 mutant, Pan‐AKT, AKT, p‐AKT, AKT2, RICTOR, RPTOR, BECN2, BAX, CASP3, AKT1, mTOR, p‐mTOR, p‐4E‐BP1, BECN1, KLC3B, CDK4, and GAPDH.	–	–	–	L
Xia et al.^30^	T24, EJ, and SV‐HUC‐1	Bladder and urothelial cancer	Control and blue laser	CCK‐8 assay, 24 and 48 h after irradiation.	Flow cytometry for cell cycle after 48 h and immunohistochemistry for Ki‐67 after 48 h.	Flow cytometry to annexin V.	WB to Ki67, CDK4, MMP2, MMP9, SNA1, CDH1, CDH2, MEK, p‐MEK, ERK, p‐ERK, H3P38, p‐H3P38, and GAPDH.	–	–	Migration assay after 0, 12, and 24 h, transwell assay for migration after 24 h and invasion after 48 h.	L
He et al.^39^	U‐2 OS and 143B	Osteosarcoma	Control, blue LED, red LED, and green LED	Trypan blue exclusion assay after irradiation.	EdU assay.	Evaluated by PI and Hoechst 33342 and tunnel test.	WB to BECN1, KLC3, p62, EGFR, pEGFR, NOX2, NOX4, CAT, and β‐ACT.	RT‐qPCR to *CASP3, CASP8, LC3*, and *BECN1*.	Fluorescence assay with MitoSOX™ Red for detection of superoxide.	Immunoprecipitation, migration assay after 0, 6, 12, 24, 48, and 72 h, transwell invasion assay after 24 h, assay using mRFP‐GFP‐KLC3 adenovirus particles for autophagy detection, transmission electron microscopy, siRNA for BECN1 and ATG7.	L
Zhuang et al.^35^	Kazumi‐1	Leukemia	Control, blue LED, red LED, infrared LED, and green LED.	–	CCK‐8 assay 24 h after irradiation.	Flow cytometry to annexin V and PI, CASP3 and CASP9 activities.	WB to CASP3, PARP, and AML1‐ETO.	RT‐qPCR to *BAX, BECN2, Bcl‐2, Bcl‐xL, CASP3, CASP9*, and *β‐ACT*.	Fluorescence assay with the DCFH‐DA probe.	Analysis of mitochondrial membrane potential.	L
Hegmann et al.^32^	RT112	Bladder	Control, LED, and LED +drug.	MTT assay after irradiation.	–	Flow cytometry with PI and Hoechst 33342.	WB to PARP, NRF2, BAX, and γH2AX.	–	Fluorescence and assay with DCFH‐DA.	Detection of ATP levels and Seahorse assay to detect O_2_ consumption during mitochondrial respiration after 1 h of irradiation and activation of glycolysis.	L
Nishio et al.^16^	B16F10	Melanoma	Control, blue LED, and red LED.	Trypan blue exclusion assay 0, 24, 36, and 72 h after irradiation.	Flow cytometry for cell cycle analysis with PI after 3 and 24 h.	Flow cytometry for annexin V and PI, CASP3 detection assay.	–	RT‐PCR to *BAX, BCL‐2, SURVIVIN, p21, MITF, TYR, MC1R*, and *GAPDH*.	Flow cytometry for evaluation of superoxide anion after 3 to 24 h and cardiolipin peroxidation assay.	Analysis of mitochondrial membrane potential with DiOC6 after 3 to 24 h, chromatin condensation assay after irradiation and comet and DNA damage assay.	L
Takeuchi et al.^2^	SYO‐1, HS‐SY‐II, Aska‐SS, Yamato‐SS, and HEK293.	Synovial sarcoma	Control, blue LED, red LED, and green LED.	CCK‐8 assay 0, 24, 48, and 72 h after irradiation.	Flow cytometry for cell cycle analysis and colony formation after 24 h.	Flow cytometry for annexin V and PI.	WB for PARP, cleaved PARP, KLC3‐I/KLC3‐II, and caspase activity assay.	Microarray after irradiation for 48 h.	Fluorescence assay with CellROX and MitoSOX probe and RT‐qPCR for HMOX1, OSGIN1, and NQO1 genes.	Migration assay after 0, 24, 48, and 72 h, transwell assay and autophagy assay with CYTO‐ID fluorescent probe.	L
Zhou et al.^13^	B16F10	Melanoma	Control and blue LED.	MTT assay and trypan blue exclusion assay 24 h after irradiation.	Growth curve by cell count.	Fluorescence for apoptosis and necrosis after 24 h.	–	RT‐qPCR to the genes *MITF, TYR, TYRP1*, and *DCT*.	Fluorescence assay with the DCFDA probe for oxidation detection.	Migration assay after 0 and 18 h and melanin content assay after 3 days of irradiation.	L
Yang et al.^14^	B16F10	Melanoma	Control, blue LED, vitamin C, and blue LED + Vitamin C.	CCK‐8 assay after treatment.	–	–	WB to SVCT2, T–p‐65, N‐p‐65, and MHC class I.	RT‐qPCR to the genes *SLC23A1, SLC23A2*, and *MHC class I*.	Fluorescence assay with the DCFH‐DÁ probe for ROS detection and analysis of GPX4 and GSSG levels.	Intracellular Fe2+ detection assay, melanin detection assay.	L
Jiang et al.^37^	A549	Lung	Control and blue LED.	CCK‐8 assay 24 h after irradiation.	EdU assay after irradiation, flow cytometry (PI) for cell cycle analysis.	–	Wb to p‐TP53, TP53, p‐MAPK8, MAPK8, and β‐Actin.	RT‐qPCR to the genes *PIK3CA, AKT, ASK1, MKK4, MAPK8, TP53, BAX, BECN2, CAS3* and *β‐ACT*.	Fluorescence assay with the DCFH‐DA probe for oxidation detection.	Mitochondrial membrane potential analysis, migration assay after 0, 24, 48, and 72 h, fluorescence assay for membrane potential analysis after irradiation and LDH assay after 24 h of irradiation.	L
Teng et al.^3^	SMMC‐7721 and HepG2	Hepatic	Control and blue LED	CCK‐8 assay 24 h after irradiation.	–	Flow cytometry for annexin‐V, Muse Mitopotential kit, fluorescence with Hoechst 33342.	WB for Bcl‐2, Bax and Bad detection.	–	Fluorescence assay with the DCFH‐DA probe for oxidation detection.	Mitochondrial membrane potential analysis, migration assay after 0, 24, 48, and 72 h, transwell assay, cell membrane permeability after 24 h.	L
Silva et al.^22^	MCF‐7 and MDA‐MB‐231	Breast	Control, blue LED, red Laser, Laser+blue LED.	WST‐1 assay 48 h after irradiation.	–	–	–	–	–	Migration assay after 0 and 48 h and invasion assay with transwell.	L
Oh et al.^4^	A20, A549, HCT116, HepG2, Mia PaCa‐2, PANC‐1 and RAMOS	Lung, colorectal, hepatic, pancreatic, Burkitt's lymphoma	Control and blue LED.	Ez‐CyTox kit 48 h after irradiation.	Ez‐CyTox kit after 48 h and cell cycle analysis by flow cytometry (PI) after 10 days.	–	WB for PCNA, BECN1, KLC3A/B, CDK2, CDK4, RPS6, KB2, p‐eIF4E, MMP21, and PDCD4.	RT‐qPCR to *CASP3 and BAX* and PCR array for genes associated with metastasis.	–‐	Invasion assay with transwell and spheroid formation assay.	L
Wang et al.^33^	HepG2 and Hep3B	Hepatocellular	Control and blue LED.	CCK‐8 assay and trypan blue exclusion assay 24 h after irradiation.	EdU assay, formation of colonies and spheroids assays.	Staining assay performed with PI/Hoechst 33342 after 3 days.	WB f γ‐H2AX and β‐actina and imunofluorescence for γ‐H2AX.	–	–	Invasion assay after 0 and 24 h.	L
Farias et al.^24^	MCF‐7 and MDA‐MB‐231	Breast	Control, blue LED, red laser, blue LED + red laser.	–	–	–	–	RT‐qPCR for ACTβ, APTX, POLβ and PCNA 48 h after irradiation.	–	–	L
Sturm et al.^31^	BFTC‐905, RT‐11 and, SW‐1710	Bladder	Control, gentamicin, riboflavin, blue light and blue light+gentamicin+ riboflavin.	MTT assay 24 h after treatment and Cell Titer Blue kit assay.	–	Immunofluorescence for Hoechst 33342, PI and FDA (Fluorescein Diacetate).	–	–	Lipid peroxidation assay after 24 h.	Detection of ATP levels 24 h after irradiation, parameters relate to mitochondrial respiratory chain.	L
Yoshimoto et al.^17^	HCT‐116 co‐cultures with THP‐1 (TAMs)	Colon	Control, blue LED and M0 macrophages.	–	–	–	–	RT‐qPCR for *OPN3, CD163, CD206, PD‐L1*.	–	Assays of migration after 24 h and wound and VEGF secretion.	L
Yang et al.^40^	PAM 212 L929	Skin	Control and blue light.	–	–	–	–	–	Antioxidant N‐acetylcysteine (NAC).	Investigation of Fe(II) changes in mitochondria.	L
Jiang et al.^29^	SCC‐25	Oral squamous cell carcinoma	Control and blue light	CCK‐8 24 h after irradiation.	EdU assay 24 h after irradiation and cell cycle analysis.	Flow cytometry with annexin V‐FITC and PI.	WB for CASP‐3 (cleaved), PARP, LC3‐I, LC3‐II, Beclin‐1, and Bcl‐2.	RT‐qPCR and RNA Sequencing	Assay with DCFH‐DA probe 30 min and 24 h after irradiation.	Wound assay after 24 h, mitochondrial membrane potential assay 30 min and 24 h after irradiation. DiO, MitoTracker Red CMXRos, Hoechst 33342, ATP production.	L
Qin et al.^15^	MeWo	Melanoma	Control and blue light.	CCK‐8 assay	–	Hoechst assay kit.	–	RT‐qPCR for SOD1, CAT, BECN1, PI3K, PTEN, ASK1, P53, MMP2, SOCS3, AKT, BCL2, and RIP1.	Fluorescence probe (DCFH‐DA).	Cell cycle analysis by flow cytometry; MMPs measurement with TMRE Kit; Lactate dehydrogenase release assay with LDH kit; Caspase‐3 activity assay kit.	L
Yang et al.^38^	HOS and MG63	Osteosarcoma	Control and blue light.	CCK‐8 assay 24 h after irradiation.	Cell cycle by flow cytometry.	Flow cytometry with annexin V‐FITC and PI.	Wb analysis to PTGS2, GPX4, SLC7A11, NOX2, β‐Actin.	Transcriptome (Illumina HiseqTM 2500/4000 platform) with DEGs analysis and validation via RT‐qPCR of genes Caspase 3, *ATG13, FTH1, SLC3A2, GCLC, FTL, MYL9, FN1, COL12A1, GCLM, CPA4, ID1, CAV1*.	Fluorescence probe (DCFH‐DA).	MMP measurement with TMRE; Ferroptosis detection (Calcein‐ AM assay kit); cell migration by scratch assay.	L
Jiang et al.^28^	SCC‐25	Oral squamous cell carcinoma	Control and blue light	CCK‐8 assay 24 h after irradiation.	EdU assay 44 h after irradiation.	Flow cytometry annexin‐FITC and PI 24 h and 48 h after irradiation	–	Transcriptome – Differentially expressed genes (DEGs) and RT‐qPCR (*HSPA5, XBP1, EIF2AK3, NFE2L2, DDIT3, GADD45a, AHR, CYP1A1, CYP1B1, β‐Actin*)	Fluorescence DCFH‐DA 3 h after irradiation.	–	L
Ibrahim et al.^25^	MCF‐7	Breast	Control, blue laser, red laser and infrared laser	WST‐1 – ELISA	–	–	–	–	–	–	L
Farias et al.^23^	MCF‐7 and MDA‐MB‐231	Breast	Control group, red laser and blue LED	–	–	–	–	RT‐qPCR to genes *ACTβ, TRF1, TRF2* 48 h after irradiation.	–	Telomere length analysis by qPCR to 36B4, TEL1, TEL2.	L
Zhao et al.^41^	TPC‐1	Papillary thyroid carcinoma	Control group, red, green and blue LED	CCK‐8 assay 24 h after irradiation.	CCK‐8 assay 24 h after irradiation.	–	Wb to evaluate the retinal‐OPN3 complex: CDK4, p21, OPN3, cyclin D, cyclin E, and β‐actin.	RT‐qPCR to evaluate the retinal‐OPN3 complex: p21, *HMOX1, DHRS3, OPN3* siRNA, *OPN1SW, RHOOPN2 (OPN2), OPN3, OPN4, OPN5, OE‐OPN3*.	–	Transwell migration assay and Scratch assay for migration analysis 24 h after irradiation; cell cycle analysis by flow cytometry (PI).	L

Abbreviations: ACTβ, β‐actin; AKT, Protein kinase B; AML1‐ETO, RUNX1‐RUNX1T1 fusion protein; APTX, Aprataxin; ASK1, Apoptosis signal‐regulating kinase 1; ATG5/7/13, Autophagy‐related genes 5/7/13; ATP, Adenosine triphosphate; BAX, BCL2‐associated X protein; BECN1/2, Beclin‐1/2; BCL2, B‐cell lymphoma 2; Bcl‐xL, B‐cell lymphoma‐extra large; BrdU, Bromodeoxyuridine; CASP3/8/9, Caspases 3, 8, 9; CAT, Catalase; CCK‐8, Cell Counting Kit‐8; CDH1/2, E/N‐cadherin; CDK2/4, Cyclin‐dependent kinases 2/4; cDNA, Complementary DNA; DAPI, 4′,6‐diamidino‐2‐phenylindole; DCF‐DA/DCFDA, 2′,7′‐Dichlorofluorescin diacetate; DCT, Tyrosinase‐related protein 2; DDIT, DNA Damage Inducible Transcript 3; DEGs, Differentially expressed genes; DHRS3, Dehydrogenase/reductase 3; DiOC6, 3,3′‐Dihexyloxacarbocyanine iodide; DNA‐PK, DNA‐dependent protein kinase; EGFR, Epidermal growth factor receptor; EIF2AK3, Eukaryotic translation initiation factor 2‐alpha kinase 3; ERK/ERK1/2, Extracellular signal‐regulated kinases 1/2; FDA, Fluorescein diacetate; FITC, Fluorescein isothiocyanate; FN1, Fibronectin 1; FOXO, Forkhead box O; Fas, Death receptor Fas; GADD45a, Growth arrest and DNA‐damage‐inducible alpha; GAPDH, Glyceraldehyde‐3‐phosphate dehydrogenase; GCLC/GCLM, Glutamate‐cysteine ligase catalytic/modifier subunit; GPX4, Glutathione peroxidase 4; GSSG, Oxidized glutathione; HEK293, Human embryonic kidney 293 cells; HMOX1, Heme oxygenase 1; HSPA5, Heat shock protein family A (Hsp70) member 5; ID1, Inhibitor of DNA binding 1; IL, Interleukin; JNK, c‐Jun N‐terminal kinase; KLC3, Kinesin light chain 3; LASP1, LIM and SH3 domain protein 1; LDH, Lactate dehydrogenase; MAPK8, Mitogen‐activated protein kinase 8; MC1R, Melanocortin 1 receptor; MEK/p‐MEK, Mitogen‐activated protein kinase kinase/phosphorylated MEK; MHCI, Major histocompatibility complex class I; MITF, Microphthalmia‐associated transcription factor; MKK4, MAPK kinase 4; MMP2/9/21, Matrix metalloproteinases 2/9/21; mTOR/p‐mTOR, Mammalian target of rapamycin/phosphorylated; MTT, 3‐(4,5‐dimethylthiazole‐2‐yl)‐2,5‐diphenyltetrazolium bromide; NAC, N‐acetylcysteine; NF‐κB/NFKB, Nuclear factor kappa B; NFE2L2, Nuclear factor erythroid 2‐related factor 2; NOX2/4, NADPH oxidase isoforms; NRF2, Nuclear factor erythroid 2–related factor 2; OE‐OPN3, Opsin 3 overexpression; OPN3, Opsin 3; OSGIN1, Oxidative stress‐induced growth inhibitor 1; PARP, Poly(ADP‐ribose) polymerase; PCNA, Proliferating cell nuclear antigen; PI, Propidium iodide; PI3K/PIK3CA, Phosphoinositide 3‐kinase/catalytic subunit alpha; PLS3, Plastin 3; POLβ, DNA polymerase beta; PTEN, Phosphatase and tensin homolog; PTGS2, Prostaglandin‐endoperoxide synthase 2; qPCR/RT‐qPCR, Quantitative reverse transcription PCR; RIP1, Receptor‐interacting protein kinase 1; RPS6, Ribosomal protein S6; ROS, Reactive oxygen species; SDS‐PAGE, Sodium dodecyl sulfate‐polyacrylamide gel electrophoresis; SLC23A1/2, Sodium‐dependent vitamin C transporters 1/2; SLC3A2, Solute carrier family 3 member 2; SLC7A11, Solute carrier family 7 member 11; SOCS3, Suppressor of cytokine signaling 3; SVCT2, Sodium‐dependent vitamin C transporter 2; TCTP, Translationally controlled tumor protein; TdT, Terminal deoxynucleotidyl transferase; TEL1/2, Telomere repeat‐binding factors 1/2; TNFα, Tumor necrosis factor alpha; TP53/p‐TP53, Tumor protein p53/phosphorylated; TRF1/2, Telomeric repeat‐binding factor 1/2; TRP‐1, Tyrosinase‐related protein 1; TYR/TYRP1, Tyrosinase and tyrosinase‐related protein 1; T–p‐RELA/N‐p‐RELA, Phosphorylated RELA subunit of NF‐κB in cytoplasm/nucleus; UV, ultraviolet; VEGF, Vascular endothelial growth factor; WB, Western blot; WST‐1, Water‐soluble tetrazolium salt; ZNRF2, Zinc and ring finger 2 protein. L, Low risk of bias.

Other analyses were performed by different studies to evaluate other parameters associated with the therapeutic potential of BL. Among them, the mitochondrial membrane potential, invasion assays, migration assays, electrophoresis, spectrometry, immunohistochemistry, fluorescence, immunoprecipitation, ATP production, Seahorse, chromatin condensation, melanin content analysis, iron detection in mitochondria, LDH, and membrane permeability were analyzed.

### In vivo

All studies used rodent animals, most of which were female Balb/c mice. The age of the animals varied between 4 and 44 weeks, with the majority using rodents in the 6th week of life (Table 2). The inoculation of tumor cells into animals was predominantly performed subcutaneously^2^, ^14^, ^27^, ^34^, ^40^, ^42^ followed by the intravenous inoculation^4^, ^6^, ^20^, ^36^ and one study performed submucosal inoculation.^18^

**TABLE 2:** Summary of key characteristics of in vivo studies.

Author	Animal model	Cancer type	Specie	Age (weeks)	Sample size	Groups	*Inoculation*	Variables evaluated		
								Tumor size	Survival	Others
Ohara et al. (2002)^6^	HL60	Leukemia	Female rats Hos®:Donryu	11	Total: 100 Used:75	Control and blue LED	Daily oral administration of 0.04% ENU for 6 weeks.	Measurement 4 and 7 days after irradiation.	−	−
Ohara et al. (2003)^42^	TG‐AC mouse transduced with the v‐Ha‐ras gene	Skin	Transgenic female rats v‐Ha‐ras	44	Not informed	Control, blue LED, TPA and TPA+ blue LED	TPA application on the dorsal skin 2× per week for 9 weeks.	Measurement 1× per week for 9 weeks.	+	H&E staining, assessment of animal weight twice a week. Evaluation of erythrocytes, leukocytes and blood biochemistry to assess AST, ALT, BUN and Cre levels.
Patel et al. (2014)^27^	A431	Oral squamous cell carcinoma	Nude mice	4–6	10 (5 in each group)	Control and blue LED	Subcutaneous in the abdominal region.	Measurement on days 1, 3, 5, 8, and 12 after treatment.	−	WB for analysis of PCNA, NRF2, NF‐ĸB expression and protein oxidation assay for DNPH detection.
Oh et al. (2016)^36^	A20	B‐cell lymphoma	Balb/c mice	6	30 (15 in each group)	Control and blue LED	Caudal vein	−	+	
Oh et al. (2017)^20^	CT‐26	Colon cancer	Balb/c mice	6	30 (15 in each group)	Control and blue LED	Caudal vein	−	−	Measurement of metastases by bioluminescence 9 days after irradiation.
Kim et al. (2020)^34^	PaCa‐2	Pancreatic cancer	BALB/c‐nu/nu mice	6	Not informed	Control and blue LED	Subcutaneous	Assessment 1× per week for 5 weeks up to reaching 700–1000 mm^3^.	+	H&E staining, immunohistochemistry for AKT2, WB for AKT1/2 and weight assessment 3× per week.
Takeuchi et al. (2023)^2^	SYO‐1	Synovial sarcoma	Balb/c nude mice	4	16 (8 in each group)	Control and blue LED	Subcutaneous	Assessed every 2 or 3 days.	−	Assessment of body weight every 2 or 3 days, tumor weight at the end of treatment, WB for PARP and cleaved Caspase‐3, H&E staining and immunohistochemistry for Ki‐67 and cleaved Caspase‐3, TUNEL for detection of apoptosis.
Yang et al. (2023)^14^	B16‐F10	Melanoma	Balb/c mice	6	32 (8 groups: 4 in each group)	Control, VC, PEG‐Fns, VC + PEG‐Fns, blue LED, blue LED + VC, blue LED+PEG‐Fns and blue LED + VC + PEG‐Fns	Subcutaneous	Assessed daily until they reach approximately 400 mm^3^.	+	Evaluation of Fe + and Vitamin C production, immunofluorescence for p65, SVCT2 and DHE (ROS assessment), TUNEL for apoptosis detection, immunohistochemistry for MHC‐I.
Oh et al. (2023)^4^	A20	B‐cell lymphoma	Balb/c nude mice	6	12 (6 in each group)	Control and blue LED	Caudal vein	−	+	Measurement of metastases by bioluminescence 2 weeks after irradiation.
Yoshimoto et al. (2024)^17^	CT‐26	Colon cancer	Balb/c mice	4	8 (4 in each group)	Control and blue LED	Submucosa in the rectal region (surgical incision)	Measurement daily for 2 weeks.	−	Immunohistochemistry for anti‐F4/80, anti‐CD163 and anti‐PD‐L1 antibodies and immunofluorescence.
Yang et al. (2024)^40^	4 T1	Breast cancer	Balb/c nude mice	5–7	16 (8 in each group)	Control and blue light irradiation	Cutaneous	Measured every day.	−	H&E staining, immunohistochemistry for CD206, CD163, iNOS and NOS2.

Abbreviations: +, survival assessment; AKT, Phosphatidylinositol‐Dependent Protein Kinase; AST, Aspartate Aminotransferase; ALT, Alanine Aminotransferase; BUN, Blood Urea Nitrogen; Cre, Creatine; DHE, dihydroethidium; DNPH, 2,4‐dinitrophenylhydrazine; ENU, Ethylenethiourea; H&E, Hematoxylin–Eosin; iNOS, inducible nitric oxide synthase; LED, light‐emitting diode; MHC‐I, Major Histocompatibility Complex class I; NRF2, Nuclear Factor Erythroid 2‐Related Factor 2; NF‐ĸB, Nuclear Factor kappa B; PARP, poly(ADP‐ribose) polymerase; PCNA, Proliferating Cell Nuclear Antigen; PD‐L1, programmed death‐ligand 1; PEG‐Fns, Formulation combining polyethylene glycol (PEG) with fibronectins (Fns); VC, vitamin C; SVCT2, Vitamin C Sodium Transporter Type 2; TPA, 12‐O‐Tetradecanoylphorbol‐13‐acetate; TUNEL, terminal deoxynucleotidyl transferase dUTP nick end labeling; WB, western blotting;

Tumor size was the main method used to evaluate the effect of PBM with BL in vivo,^2^, ^6^, ^14^, ^17^, ^27^, ^34^, ^40^, ^42^ with periods ranging from 1 to 9 weeks; followed by survival analysis.^4^, ^14^, ^34^, ^36^, ^42^ Parameters such as morphological analysis, immunofluorescence, immunohistochemistry, protein and gene expression, hematological collection, and body weight monitoring were also performed (Table 2).

### Dosimetric parameters used for PBM with BL

The characteristics of the irradiation parameters used in the studies included in this review are described in Table 3. The range of wavelengths analyzed was between 380 nm and 640 nm, with all studies using at least one wavelength between 450 and 470 nm. The irradiance (mW/cm^2^) ranged from 0.27 to 550 mW/cm^2^; few studies did not report this data.^12^, ^14^, ^33^, ^35^, ^40^ Similarly, few studies did not provide data on exposure time^3^, ^4^, ^11^, ^19^, ^22^ which ranged from 6 to 172,800 s (48 h) in the studies that reported this data. Radiant exposure (J/cm^2^) was described by 23 studies and ranged from 0.2 to 1080 J/cm^2^.^4^, ^11^, ^13^, ^14^, ^15^, ^17^, ^19^, ^22^, ^23^, ^24^, ^25^, ^26^, ^27^, ^28^, ^29^, ^30^, ^31^, ^32^, ^33^, ^34^, ^37^, ^39^, ^40^, ^41^ Most studies opted for the whole plate irradiation protocol with a single irradiation. In all studies, the control group was not irradiated, remaining under the same experimental conditions as the other groups without irradiation. Regarding the physical characteristics of the irradiation, few studies included information on the opening diameter (cm),^26^, ^27^, ^34^ beam spot size on target (cm^2^)^22^, ^23^ and total radiant energy (J).^22^ In in vivo studies, the same light sources were used as in the in vitro model, with variations only in dosimetry and irradiation frequency. Irradiance (mW/cm^2^) ranged from 0.0041 to 500 mW/cm^2^, as analyzed by 9 of the 11 in vivo studies^2^, ^4^, ^17^, ^20^, ^27^, ^34^, ^36^, ^40^, ^42^ and the exposure time ranged from 60 to 28,880 s (8 h) according to the 10 studies that reported this parameter.^2^, ^4^, ^6^, ^14^, ^17^, ^20^, ^34^, ^36^, ^40^, ^42^ Radiant exposure (J/cm^2^) was reported in only three studies, ranging from 0.078 to 77 J/cm^2^.^14^, ^27^, ^40^ The opening diameter was documented by two studies.^27^, ^34^ The number of sessions ranged from a single session to three times per day^2^, ^17^, ^20^, ^34^, ^36^, ^40^ and the frequency of sessions varied from a single session to daily for a maximum of 36 sessions.^2^, ^17^, ^20^, ^27^, ^34^, ^36^, ^40^, ^42^ Other information, such as total radiant energy, radiant power (J), radiant energy per point (E), beam spot size on the target (cm^2^), polarization, and spectral bandwidth (nm) were not mentioned by the studies.

**TABLE 3:** Dosimetric parameters used in in vitro and in vivo studies.

Author	Laser device model	Wavelength (nm)	Irradiance (mW/cm^2^)	Exposure time (s)	Radiant exposure (J/cm^2^)	Irradiation sessions	Other information
Ohara et al.^10^	Blue LED (Nichia Corp, Tokushima)	470, 634, 518 and 253.7 nm	0.13, 2.3, 2.7, and 5.7	600, 1200, 2400 s	–	Single and 2 sessions in the 2400 s group (2× 1200 s with 1 h break).	Emission power: 30.1 mA
Ohara et al.^6^	Blue LED (Nichia Corp, Tokushima)	–	5.7	18,000, 10,800 s	–	1	Emission power: 30.1 mA
Ohara et al.^42^	Blue LED (Nichia Corp, Tokushima)	470 nm	5.7	3600 s	–	Daily irradiation for 9 consecutive weeks.	–
Lockwood et al.^26^	LED composed of quartz‐tungsten‐halogen, Optilux (SDS‐Kerr, EUA)	380–500 nm	550	6, 30, 60 s	3, 15 or 30	1	Opening diameter: 10,4 mm
Sparsa et al.^11^	Blue LED – PDT 450 L. (WALDMANN) and Red LED (PhoyoDyn 501.C)	450 and 800 nm	10	–	10 and 20	1	–
Sato et al.^12^	LED D A.S. (Tech Corp, Osaka)	465, 640, 590 and 520 nm	–	900–129,600 s	–	1	–
Patel et al.^27^	QTH Dental Curing LED (Quartz‐Tungsten‐Halogen)	400–500 nm	500	–	15 and 45	1	Opening diameter: 6.4 mm and 15.6 mm In vivo: Animals were irradiated daily with 45 J/cm^2^ for 12 days.
Matsumoto et al.^21^	LED NCSB119 (NICHIA Corporation)	465, 525, and 635 nm	15 and 30	600 s	–	Daily irradiation for 5 consecutive days.	–
Oh et al.^36^	Not informed	450 nm	0.0041 and 6.3	1800–14,400 s	–	3	In vivo*:* irradiance 0.0041 mW/cm^2^. Animals were irradiated daily for 3 days for 3 h.
Oh et al.^20^	Not informed	450 nm	6.3	600, 1800, 3600, 4140 s	–	3	In vivo: irradiance 8.23 mW/cm^2^. Animals were irradiated daily for 3 days for 3 h.
Yan et al.^19^	Not informed	470 nm	20	–	72, 144, 216, and 288	1	–
Yoshimoto et al.^18^	LED NCSB119 (NICHIA Corporation)	465 nm	30	600, 1800 s	–	1	–
Kim et al.^34^	LED skin adhesive strip (Color Seven Co.)	460 nm and 610 nm	5 and 10	10,800, 18,000 s	12	Daily irradiation for 5 consecutive days.	Aperture diameter: 4 mm In vivo: animals irradiated to 7200 s daily for 5 days.
Xia et al.^30^	Diode laser system (brand not specified)	450 nm	100	20 s‐240 s	2, 4, 8, 12, 16, 20, and 24	1	–
He et al.^39^	Not informed	470, 560 and 630 nm	100	1800–10,800 s	180, 360, 720, and 1080	1	–
Zhuang et al.^35^	Not informed	456, 515, 630 and 840 nm	–	3600, 7200, 10,800 s	–	1	–
Hegmann et al.^32^	LED Philips Research	453 ± 10 nm	39	2820 s	110	1	–
Nishio et al.^16^	LED A.S. Tech Corp., Osaka	465 nm and 640 nm	1 and 5	900–86,400 s	–	1	–
Takeuchi et al.^2^	Teleopto LED LEDA‐X LED Array with driver LAD‐1; Amuza)	470 nm, 525 nm and 639 nm	0.1 and 0.6	172,800 s (48 h)	–	12	In vivo: WS2812B equipment (WorldSemi) with irradiance of 30 mW/cm^2^ 8 h/day for 12 consecutive days was used.
Zhou et al.^13^	LED AITECSYSTEM CO., LTD.	470 nm	0,27	0, 3600, 7200 s	0, 1, and 2	Daily irradiation for 3 consecutive days.	–
Yang et al.^14^	LED Incubator Lighting ZWYC‐290 A (Zhichen, China)	450 nm	–	1200, 2400, 3600 s		1	In vivo*:* irradiation with 5 W LED lamp at 0.078 J/cm^2^
Jiang et al.^37^	Not informed	457 nm	1,3, 5, 10, 20, and 50	600, 1200, 2400, 4800 s	1,2–24	1	–
Teng et al.^3^	Irradiation system composed of 192 LEDs (brand not specified)	453, 521, 623 nm	63	–	–	1	
Silva et al.^22^	LED HTM (São Paulo, Brasil)	470 nm	5.35	–	160, 321, and 482	1	Beam spot size on target (cm^2^): 0.28 cm^2^ Power: 150 mW
Oh et al.^4^	LED BL‐irradiating device (brand not specified)	450 nm	7.0 mW/cm^2^	–	5 to 20	1	–
Wang et al.^33^	Not informed	470 nm	–	900, 1800, 2700, 3600 s	0, 72, 144, 216, and 288	1	–
Farias et al.^24^	Therapeutic Laser and Blue LED: HTM Indústria de Equipamentos Eletroeletrônicos Ltda	470, 658 nm	0.77 and 5.35 W/cm^2^	90 s	69 and 482	1	Emission power: 0.1 (red) or 0.15 W (blue) Beam size: 0.13 (red) and 0.28 cm^2^ (blue)
Sturm et al.^31^	LED device 10 cm × 12 cm containing 60 Philips Research LEDs	453 nm	39 mW/cm^2^	2820 s	110	1	Plate temperature was measured Maximum intensity: 0.21 W/nm Distance between LED and Monolayer Cell: 5 cm Optical Power: 13 W Number of LEDs: 60 LEDs
Yoshimoto et al.^17^	LED device with an array of 10 cm × 12 cm LEDs.	453 nm	30 mW/cm^2^	1800 s	54	1	In vivo: Irradiation with LED lamp NCSB119, NICHIA Corporation, Tokushima, Japan, 465 nm, 30 mW/cm^2^, 1800 s, single irradiation.
Yang et al.^40^	Light intensity was measured with a digital lx meter (SW‐582, SNDWAY). The mice and cells were irradiated in a ZWYC‐ 290A illumination incubator (ZHICHENG).	450 nm	–	180 s	231	14	In vivo*:* radiant exposure: 77 J/cm^2^, time of exposure 2 per day (1 h each) for 7 days.
Jiang et al.^29^	LightEngin, from Shanghai, China	420 nm	2.5, 5, 10, 25, 50, 70 mW/cm^2^	120 s, 240 s, 480 s, 720 s, 960 s, 1200 s	3, 6 12, 24, 30, 36, 48, and 60	1	Spectral bandwidth: 15 nm
Qin et al.^15^	Light cell incubator (Shanghai Lightengin Technology Co., Ltd., China)	457 nm (FWHM = 20 nm).	6.4; 12.8; 25.6	37.5, 75, 150, 300, 600, 1200, 2400, 4800 s	0.96; 1.92; 3.84; 7.68; 15.36; 30.72	1	**–**
Yang et al.^38^	Light incubator (Lightengin, China)	420, 460, and 480 nm	Parameters 1: 0, 0.5, 1, 2, 5, 10, 25, 35 40, 50, and 60 Parameter 2: 25	Parameters 1: 1200 s 2: 0, 600, 1200, 2400, 3600 s	**–**	1	**–**
Jiang et al.^28^	Cell irradiation incubator (LightEngin, Shanghai, China)	457 and 475 nm	2.5, 5, 10, 25, and 50	1200 s	3, 6, 12, 30, 60	1	The precise irradiance was measured using a light power. Temperature of culture medium was monitored using a thermal resistance thermometer
Ibrahim et al.^25^	Not informed	473 nm	10, 25, 45, and 65	60, 300, 600, and 900 s	3, 7.5, 13.5, 15, 19.5, 30, 37.5, 45, 67.5, 75, 97.5, 112.5, 135, 195, 202.5, and 292.5 J/cm^2^	1	Distance between the laser and well plates was 4 cm. Area where the lasers irradiated the cells was covered with black felt cloth to prevent light dispersion caused by reflections on the surfaces of the biosafety cabinet. Laser temperature: 0°C to +70°C
Farias et al.^23^	Therapeutic Laser and Blue LED: HTM Indústria de Equipamentos Eletroeletrônicos Ltda (Fluence – Brazil)	470	5.35 W	90 s	482/cm^2^	1	the pellets were exposed at a distance of 7 cm from the laser‐LED
Zhao et al.^41^	Programmable 5‐W LED lighting system (Xuzhou Ai Jia Electronic Technology Co., LTD)	465	2	86,400 s	172.8	1	Photon energy: 2.66 Einstein dose (photons): 4.05 × 10^20^

### In vitro and in vivo effects of PBM with BL

The biological effects of PBM with BL from the in vitro and in vivo studies are detailed in Table 4.

**TABLE 4:** Summary of the main findings from the in vitro and in vivo studies included in the systematic review.

Study	Results
Ohara et al.^10^	**In vitro** Viability: Blue LED irradiation reduced cell viability after 48 h proportionally to exposure time. Proliferation: Lower proliferation after blue LED irradiation and reduction in colony size, increase in the percentage of cells in G0/G1 and decrease in S after 72 h, more evident in cells that were exposed twice to blue LED. Cell death: no difference between the control and irradiated groups
Ohara et al.^6^	**In vitro** Cell viability: Reduction in the number of circulating leukemia cells after blue LED irradiation **In vivo** Cell viability: Reduction in the number of leukemia cells after 4 and 7 days of extracorporeal irradiation with blue LED.
Ohara et al.^42^	**In vivo** Inhibition of the growth of non‐melanoma skin tumors after 6, 7, and 9 weeks of TPA administration in animals irradiated with blue LED.
Lockwood et al.^26^	**In vitro** ROS generation: Increased ROS after 2 h of irradiation, depending on radiant exposure and cell type. The OSC2 strain showed high levels of ROS after irradiation with 15 J/cm^2^, which remained elevated for 2 h. In the NHEK strain, all parameters evaluated induced a slight increase in ROS, with the exception of 3 J/cm^2^, in which no effect was observed.
Sparsa et al.^11^	**In vitro** Cell viability: Reduction in the number of B16F10 cells after blue LED irradiation in all evaluated periods. No effect of irradiation on lipid peroxidation was observed with both radiant exposures evaluated as well as in the endothelial cell line tested.
Sato et al.^12^	**In vitro** Cell viability: Reduction of viability after 24 and 72 h of blue LED irradiation. Proliferation: Reduction of cell proliferation after 72 h, in all irradiation parameters tested. ROS generation: Increase in superoxide anion after 6 and 12 h of irradiation and loss of mitochondrial membrane potential after blue LED irradiation, especially in the longer irradiation times.
Patel et al.^27^	**In vitro** Viability: Reduction of viability after 24, 48, and 72 h of blue LED irradiation as well as reduction of mitochondrial activity, which was more pronounced at 45 J/cm^2^ at 72 h. There was an increase in the nuclear protein expression of NRF2 and its transcriptional target HO1 after LED irradiation, especially at 45 J/cm^2^, remaining elevated for 6 h. **In vivo** Inhibition of tumor growth after daily irradiation with blue LED, detectable from the 4th day of treatment. There was a reduction in cell proliferation in irradiated tumors as well as a reduction in ROS formation.
Matsumoto et al.^21^	**In vitro** Inhibition of tumor growth after daily irradiation with blue LED, detectable from the 4th day of treatment. There was a reduction in cell proliferation in irradiated tumors as well as a reduction in ROS formation.
Oh et al.^36^	**In vitro** Viability: Reduction of cell viability after 1, 2, 3, and 4 h of blue LED irradiation. Cell death: Significant increase in the percentage of apoptotic cells after blue LED treatment, which was accompanied by increased cleaved Capase‐3 and PARP, as well as a reduction in Bcl‐2. ROS: Increased production of superoxide anion and loss of membrane potential after 2 h of blue LED treatment. Autophagy: Increase in the LC3‐II/LC3‐I ratio after 2 and 4 h of irradiation. Blocking the initial phase of autophagy with specific inhibitor resulted in decreased LC3‐II inhibition and cleaved Caspase 3. Additionally, the inhibition of ATG5 and beclin‐1 with siRNA resulted in a decrease in the percentage of apoptotic cells, indicating that one of the mechanisms by which the blue LED induces apoptosis is via activation of autophagy pathway signaling. **In vivo** Increased survival in animals submitted to blue LED irradiation.
Oh et al.^20^	**In vitro** Viability: Reduction of cell viability after 24 and 48 h of irradiation. Migration and invasion: Decreased capacity for migration and cell invasion, associated with inhibition of MMP2 and MMP9 through p38 phosphorylation. **In vivo** Reduction of lung metastases.
Yan et al.^19^	**In vitro** Viability: Reduction of cell viability after 24 and 48 h of irradiation. Proliferation: Decrease in the percentage of positive EdU and Ki‐67 cells after blue LED irradiation. Cell death: Increased percentage of apoptotic cells, with increased Bax expression and decreased Bcl‐2 expression. ROS: Increased ROS formation in the nucleus and cytoplasm after irradiation as well as γH2A.X. expression, indicating DNA damage. Migration: Decreased cell migration capacity after 48 and 72 h of irradiation, with increased expression of E‐cadherin and reduction in the expression of N‐cadherin, vimentin and TWIST, associated with inhibition of MMP2 and MMP9 through p38 phosphorylation.
Yoshimoto et al.^18^	**In vitro** Viability: Reduction of cell viability after 48 h of blue LED irradiation for 30 min. Cell death: Increased gene expression of Caspase‐8. Autophagy: Increased levels of LC3‐II and Beclin‐1 mRNA and protein and autophagosomes. siRNA: Silencing of the Opn3 gene was able to reverse the antitumor effects evidenced by irradiation with the blue LED.
Kim et al.^34^	**In vitro** Viability: Decrease in cell viability proportional to dose and irradiation time with the blue LED. Proliferation: Reduction in the percentage of BrdU‐positive cells and clonogenic potential. There was an increase in irradiated cells in the G0/G1 phase of the cell cycle in one of the evaluated cell lines and in the expression of CDK4 and cyclin D1. Cell death: Increase in the percentage of apoptotic cells and decrease in Bcl‐2 expression with an increase in Capsase‐3 and cleaved PARP in one of the evaluated strains. Induction of apoptosis was associated with inhibition of the Akt/mTOR pathway due to decreased phosphorylation of AKT, mTOR **In vivo** Decreased tumor growth after blue LED treatment and reduced AKT2 expression.
Xia et al.^30^	**In vitro** Viability: Decrease in cell viability proportional to the dose of irradiation with the blue laser, with an inhibitory effect observed from 4 J/cm^2^. Proliferation: Reduction in the number of Ki‐67 positive cells. No effect was observed in relation to cell cycle and CDK4 expression. Decrease in p‐MEK and p‐ERK. Migration and invasion: Inhibition of cell migration and invasion after 24 and 48 h of irradiation, respectively, as well as inhibition of MMP2 and MMP9 expression. There was an increase in the expression of E‐cadherin and a decrease in the expression of Snail and N‐cadherin. Inhibition of the MPK/MEK/ERK pathway after blue laser irradiation.
He et al.^39^	**In vitro** Viability: Decreased cell viability and radiant exposure above 360 J/cm^2^ does not promote further reduction in viability. Proliferation: Inhibition of cell proliferation with radiant exposure above 360 J/cm^2^. Cell death: Increased percentage of apoptotic cells. ROS: Increased ROS production after blue LED irradiation and NOX2 and NOX4 expression Autophagy: Induction of autophagy after treatment with blue LED, with increased presence of autophagosomes and autophagic vesicles, protein expression of Beclina‐1 and LC3‐II/LC3‐I ratio. Treatment with autophagy inhibitor was able to reverse the antitumor effects of blue LED irradiation. In addition, the activation of autophagy by the blue LED occurs through the EGFR/Beclina‐1 pathway. Migration and invasion: Inhibition of migration and invasion in all periods evaluated.
Zhuang et al.^35^	**In vitro** Proliferation: Decrease in cell proliferation in proportion to the time of irradiation. Apoptosis: Increased percentage of apoptotic cells and Bax gene expression, as well as reduced Bcl‐xL and Bcl‐2 mRNA levels. There was an increase in Caspase‐3, PARP, and AML1‐ETO protein after treatment with blue LED. ROS: Increased production of ROS accompanied by reduced mitochondrial membrane potential.
Hegmann et al.^32^	**In vitro** Cell viability: Decreased viability only when blue LED irradiation was associated with mitomycin C. Cell death: Increased percentage of apoptotic cells and cleaved Bax and PARP expression. ROS: Increased production of ROS, reduced production and consumption of ATP, reduced glycolysis rate, glycolytic capacity and reserve.
Nishio et al.^16^	**In vitro** Cell viability: Decrease in viability proportionally to the irradiation time. Proliferation: Decreased cell proliferation after 72 h of blue LED irradiation and increased percentage of cells in G2/M of the cell cycle after 24 h Cell death: Increase in the percentage of apoptotic cells according to increased irradiance, Caspase‐3 activation, greater chromatin condensation, and direct DNA damage when cells were irradiated for 12–24 h. There was an increase in the gene expression of Survivin as well as a reduction in the expression of p21. ROS: Increased ROS production from 6 h of irradiation and lipid peroxidation, loss of mitochondrial membrane potential with 12 and 24 h of irradiation.
Takeuchi et al.^2^	**In vitro** Cell viability: Decrease in viability proportional to irradiation time and irradiance. Cell death: Higher rate of apoptosis, higher expression of genes associated with apoptosis, and intrinsic pathway of apoptosis activation in microarray analysis, induction of PARP and Caspase expression 3/7. Migration and invasion: Reduction of migration and invasion after LED irradiation and negative correlation between the expression of genes associated with metastasis and blue LED treatment. ROS: Increased production of ROS in the mitochondria after blue LED irradiation for 48 h, enrichment of genes associated with ROS formation, mitochondrial ROS formation is responsible for blue light‐induced apoptosis since the use of ROS inhibitor reversed the antitumor effects of blue LED. Autophagy: Induction of autophagosome formation by blue light according to the increase in exposure time, increase in LC3B‐II and PARP, siRNA for LC3B promoted inhibition of cell growth the expression of PARP and the use of autophagy inhibitor associated with irradiation was able to potentiate the antitumor effects observed by the blue LED. **In vivo** Inhibition of tumor growth, with the formation of smaller tumors. There was an increase in the protein expression of cleaved PARP and Caspase‐3 in the group treated with blue LED.
Zhou et al.^13^	**In vitro** Cell viability: Decrease in viability proportionally to the irradiation time. Cell death: No difference in the rate of apoptosis. Migration: Decreased migration capacity. ROS: Increase in ROS formation and DNA damage proportional to irradiation time. Increased expression of genes associated with melanin synthesis.
Yang et al.^14^	**In vitro** Cell viability: No effect of blue LED irradiation on viability was observed when applied alone. The combination of blue LED + vitamin C resulted in reduced viability. Cell death: Synergistic effect of blue light with Vitamin C, increasing oxidative stress and inducing ferroptosis. ROS: Increasing ROS formation through activation of the NFkB pathway The blue LED increases the uptake and amount of intracellular vitamin C. **In vivo** The blue light effectively penetrated the skin of the mice, reaching the melanoma. The blue LED combined with vitamin C resulted in increased ROS production via activation of the NFkB pathway, resulting in the accumulation of vitamin C in tumor tissue. The combination of treatment with blue LED + vitamin C + PEG‐Fns promoted inhibition of tumor growth.
Jiang et al.^37^	**In vitro** Viability: Decreased cell viability with 3 mW/cm^2^ and 3.6 J/cm^2^. Proliferation: Blue LED induced cell cycle arrest in G2. Gene expression: Reduced expression of PI3K and AKT and increased gene expression of p53, ASK1, MKK4, and JNK, as well as phosphorylation of p53 and JNK. ROS: Increased ROS formation and loss of mitochondrial membrane potential. Migration: Inhibition of cell migration after blue LED irradiation.
Teng et al.^3^	**In vitro** Viability: Decreased cell viability after 24 h of blue LED irradiation Proliferation: Inhibition of cell proliferation after blue LED irradiation, proportional to the irradiation time. Cell death: Increase in apoptotic cells and evident nuclear change after blue light treatment, increase in Bax and Bad proteins, and reduction in Bcl‐2 protein ROS: Increased ROS formation and loss of mitochondrial membrane potential. Migration and invasion: Inhibition of cell migration and invasion from 24 h after irradiation
Silva et al.^22^	**In vitro** Viability: There was no effect on cell viability after 48 h of blue LED irradiation. Migration and invasion: Blue light did not alter the migratory capacity of the evaluated strains, but inhibited cell invasion with radiant exposure of 482 J/cm^2^.
Oh et al.^4^	**In vitro** Proliferation: Inhibition of cell proliferation after 24 and 48 h of irradiation with 10 and 20 J/cm^2^, reduction in the percentage of cells in the S phase of the cell cycle and slight increase in the number of cells in the G0/G1 phase, reduction in the expression of the protein PCNA and increase in the expression of CDK2, CDK4, Beclin‐1, and LC3A/B. A synergistic effect of blue light with the chemotherapy drugs 5‐FU and gentamicin was observed in relation to cell proliferation and in the size of the spheres with the association of the LED with the 5‐FU. Invasion: Inhibition of cell invasion after irradiation. **In vivo** Animals irradiated with blue LEDs showed increased survival and reduced metastasis formation, as well as decreased gene expression of CCL7, HPSE, MMP10, MYC, SET, SRC, SYK, and TGFβ1.
Wang et al.^33^	**In vitro** Cell viability: Decrease in viability proportional to the radiant exposure used. Proliferation: Inhibition of cell proliferation and reduction in the number of tumor spheres. Irradiation with blue LED potentiated the antiproliferative effect of the chemotherapy drug sorafenib and reduced the number of colonies. Cell death: increase in the percentage of dead cells and apoptotic cells. Increased percentage of γ‐H2AX‐positive cells, indicating DNA damage caused by blue light. Migration and invasion: inhibition of cell migration in all periods evaluated and cell invasion after 24 h of irradiation.
Farias et al.^24^	*In vitro:* Gene expression: Blue LED reduced mRNA levels for APTX in MDA‐MB‐231. Both types of irradiations resulted in significant reductions in mRNA levels for Polβ and PCNA, but the blue LED had a comparable or greater impact, especially for APTX in MDA‐MB‐231.
Sturm et al.^31^	*In vitro:* Viability: Blue light alone caused a significant reduction in cell viability at parameters 140, 200, and 300 J/cm^2^, but the effect was less pronounced than when combined with riboflavin. Cell death: Blue light induced an increase in cell death, evidenced by an increase in the percentage of apoptotic and necrotic cells. However, the effect was smaller compared to the combination of blue light with riboflavin and/or gemcitabine. ROS: Exposure to blue light alone increased lipid peroxidation, although to a lesser extent than combination with riboflavin as well as reduced ATP production and maximal mitochondrial respiration.
Yoshimoto et al.^17^	**In vitro** Migration and invasion: There was a reduction in the migration capacity of tumor cells when incubated with conditioned macrophage medium submitted to blue light irradiation. Gene expression and protein secretion: Blue LED irradiation decreases the expression of CD206 and CD163 as well as the secretion of VEGF in macrophages, indicating a possible inhibitory effect on the polarization of these cells. **In vivo** Blue LED irradiation reduced tumor growth, PD‐L1 expression, and the frequency of F4/80 and CD163‐positive cells compared to the control.
Yang et al.^40^	**In vitro** blue light irradiation increased intracellular ROS **In vivo** Blue light irradiation increased intracellular ROS. Low‐intensity epidermal blue light‐irradiated mice showed stimulated innate immunity, which was able to decrease subcutaneous tumor volume and pulmonary metastatic tumor nodules through activated macrophages and neutrophils.
Jiang et al.^29^	**In vitro** Cell Viability: Viability was reduced proportionally to the increase in irradiance. Exposure to 50 mW/cm^2^ caused a significant increase in culture temperature, which impacted cell viability, while exposures to 25 and 5 mW/cm^2^ kept cell viability within acceptable limits. Cell Death: Exposure to blue light induced apoptosis in SCC‐25 cells. The number of apoptotic cells increased significantly at 24 and 48 h after irradiation, with an increase in the population of cells in early and late apoptosis. Proliferation: Cell proliferation was inhibited by blue light, as evidenced by the EdU assays. The number of dividing cells decreased with increasing irradiation dose. Protein Expression: Exposure to 420 nm blue light resulted in an increase in endoplasmic reticulum stress‐associated proteins and NF‐κB and p53 proteins. Gene expression: Exposure to blue light led to a differential regulation of 154 down‐regulated and 316 up‐regulated genes, with emphasis on increased endoplasmic reticulum stress and apoptosis. ROS: The level of intracellular ROS increased after exposure to 420 nm blue light, showing a persistent pattern of change over time. The expression of ER stress‐related genes, identified by RNAseq and confirmed by RT‐qPCR, also indicated a significant increase in response to blue light treatment.
Qin et al.^15^	**In vitro** Cell viability: blue light exerted an inhibitory effect on MeWo cells, with a discernible dose threshold, intensifying with increasing doses. Apoptosis: Blue light induced apoptosis and led to cell cycle arrest. ROS: ROS level was not changed. Gene expression: SOD, CAT, BECN1, PTEN, ASK1, P53, MMP, and AKT were immediately upregulated upon blue light treatment, while BCL2 and RIP1 downregulated and ASK1 was kept upregulated. SOD, P53, MMP2, and AKT transitioned from their immediate post‐treatment upregulation to downregulation. CAT, BECN1, and PTEN were no longer upregulated. BCL2, which was downregulated immediately after treatment, transitioned to an upregulated state, RIP1 was no longer downregulated. Both PI3K and SOCS3 exhibited upregulation at this time. Others: Blue light inhibited MMP and increased caspase‐3 levels.
Yang et al.^38^	**In vitro** Cell viability: decreased in a manner dependent on both the wavelength of blue light and the duration of exposure. Apoptosis: varied blue light wavelengths triggered cell death – exposure to 420 nm blue light significantly elevated the apoptosis index of HOS and MG63 cells by 2.5 and 2.8 times, respectively; under 460 and 480 nm, apoptosis was slightly increased. ROS: Blue Light PBM stimulated ROS generation and induced MMP loss. Protein and Gene expression: differential expression of protein and genes associated with ferroptosis, oxidative stress, and iron metabolism. Cell migration: blue light inhibited cell migration and induced cell cycle arrest. Others: Blue light induced ferroptosis.
Jiang et al.^28^	**In vitro** Cell viability: Both blue light wavelengths (457 and 475 nm) suppress cell viability of SCC‐25 cells (higher in 457 nm). Apoptosis: irradiation with both 457 and 475 nm LEDs did not result in an increase in apoptotic cells. Proliferation: Both 457 nm and 475 nm LED irradiation led to an increase in G2/M phase cells and a decrease in G1 phase cells (higher in 457 nm). Proliferation decreased 24 h after irradiation. A decrease in the proportion of EdU+ cells was observed in cells treated with 457 nm light 48 h after irradiation, however, it returned to normal levels in cells irradiated with 475 nm light. ROS: Both 457 and 475 nm blue light exposure induced the ROS production in SCC‐25 cells. Gene expression: Genes associated with ER stress, HSPA5, XBP1, EIF2AK3, DDIT3, NFE2L2, and GADD45a were significantly upregulated after 457 nm light exposure, while only XBP1 and GADD45a showed significant upregulation after 475 nm light exposure. Genes associated to AHR pathway, CYP1A1, and CYP1B1 significantly increased expression levels under 457 nm, whereas under 475 nm light exposure, only CYP1A1 exhibited significant upregulation.
Ibrahim et al.^25^	**In vitro** Cell viability: blue laser showed a high cell survival rate and a low influence on MCF‐7 breast cancer cell lines. Blue laser demonstrated increased cell viability with higher power, but this effect decreased as the exposure duration extended.
Farias et al.^23^	**In vitro** Gene expression: Blue LED decreased the TRF1 and TRF2 mRNA levels in breast cancer cells. Telomere length was increased in MCF‐7 cells after exposure to blue LED. However, telomere length in MDA‐MB‐231 was shortened after exposure to blue LED.
Zhao et al.^41^	**In vitro** Cell viability and proliferation: Blue light exposure led to the dissociation of 11‐cis‐retinal from OPN3, resulting in the accumulation of all‐trans retinal which disrupted cellular proliferation pathways and induced G0/G1 cell cycle arrest in PTC cells. Migration and invasion: BL inhibited migration and invasion of PTC cells Gene expression: decrease in Dehydrogenase/Reductase member 3 (DHRS3), critical for converting retinal to retinol; sharp decrease in NOX5 directly affected ROS and impeded cell cycle progression levels ROS: increased ROS levels significantly reduced the efficacy of blue light PBM

#### In vitro

##### Cellular viability

Across 28 studies,^2^, ^3^, ^4^, ^6^, ^10^, ^11^, ^12^, ^13^, ^14^, ^15^, ^16^, ^19^, ^20^, ^22^, ^25^, ^27^, ^28^, ^29^, ^30^, ^31^, ^32^, ^33^, ^34^, ^36^, ^37^, ^39^, ^40^, ^41^ BL therapy consistently induced a dose‐ and time‐dependent reduction in cell viability (Table 4). Increasing the irradiation time has been shown to be one of the main contributing factors to this decrease in viability, occurring mainly 24 and 48 h after irradiation. Three studies^14^, ^25^, ^32^ found no effects on cellular viability under BL alone, but reduced viability was noticed when BL was associated with mitomycin C^32^ and vitamin C.^14^ In addition, Ibrahim et al. (2024) observed an increase in cell survival after BL irradiation, but this effect decreased as the exposure duration extended.^25^

##### Cellular proliferation

Cell proliferation was evaluated in 19 studies^2^, ^4^, ^10^, ^12^, ^13^, ^16^, ^18^, ^19^, ^21^, ^27^, ^28^, ^29^, ^30^, ^33^, ^34^, ^35^, ^37^, ^39^, ^41^ and a reduction in proliferation was observed after irradiation with BL, even in the face of the methodological diversity and dosimetric parameters used (Table 4). Studies that evaluated the clonogenic potential have consistently identified a reduction in the number and size of colonies.^10^, ^34^ In the study of Wang et al. (2023), BL potentiated the effect of chemotherapy in hepatic carcinoma, resulting in a further decrease in the number of colonies.^33^

Regarding the cell cycle, several changes were observed, such as an increase in the percentage of cells in the G0/G1 phase,^4^, ^10^, ^34^ a decrease or stop in the G2/M phase^37^ and a reduction in the S phase.^4^ On the other hand, Jiang et al. (2024) and Nishio et al. (2022) identified an increase in G2/M phase cells in oral squamous cell carcinoma and in melanoma, respectively. Jiang et al. (2024) also observed a decrease in the G1 phase of oral cancer cells after irradiation with both 457 and 475 nm. Zhao et al. (2024) noted that BL induced G0/G1 cell cycle arrest in papillary thyroid carcinoma cells, while Matsumoto et al. (2014) observed a decrease in both G0/G1 and G2/M phases after irradiation of colon cancer cells.^16^, ^21^, ^28^ Studies have shown a reduction in the protein expression of PCNA^4^ and an increase in the expression of CDK2, CDK4, Beclin‐1, LC3A/B, and cyclin D1,^4^, ^34^ indicating an inhibition of cell cycle progression, cell survival signaling, and cell proliferative activity.

Studies that performed gene expression analyses of genes involved in the activation of signaling pathways associated with cell proliferation identified decreased *ERK1/2* mRNA levels,^21^ *p‐MEK* and *P‐ERK*,^30^ and an increase in the gene expression of *p53*, *ASK1*, *MKKA*, and *JNK*, as well as in the phosphorylation of *p53* and *JNK* and *Opn3*.^17^, ^24^, ^37^

##### Cell death

The effects of BL in cell death were evaluated by 20 studies^2^, ^3^, ^10^, ^13^, ^14^, ^15^, ^16^, ^18^, ^19^, ^21^, ^28^, ^29^, ^31^, ^32^, ^33^, ^34^, ^35^, ^36^, ^38^, ^39^ and, with the exception of only three studies,^10^, ^13^, ^28^ a pro‐apoptotic effect of BL was observed, with an increase in the percentage of apoptotic cells and in the expression of apoptosis‐related proteins such as Caspase‐3 and PARP,^2^, ^16^, ^19^, ^21^, ^34^, ^35^, ^36^ Caspase‐8,^18^, ^21^ Bax,^3^, ^19^, ^32^ Bad^3^ Survivin,^16^ P21,^16^ Caspase‐7^2^ and AML1‐ETO.^35^ On the other hand, it was observed that there was a reduction in the expression of proteins Bcl‐2,^19^, ^34^, ^35^ Bcl‐xL^35^ and p21.^16^ Only three studies did not identify any differences in apoptosis rates when compared to the control group^10^, ^13^, ^28^ (Table 4).

##### Production of Reactive Oxygen Species

Increased ROS production was observed in 19 studies^2^, ^3^, ^12^, ^13^, ^14^, ^15^, ^16^, ^19^, ^26^, ^28^, ^29^, ^31^, ^32^, ^35^, ^36^, ^37^, ^38^, ^39^, ^40^, ^41^ being variable according to the irradiation parameter and cell type (Table 4). In general, studies observed an increase in ROS levels associated with a decrease in the membrane potential. These findings suggest significant implications in cellular physiology, as ROS can damage DNA and other biomolecules specific to cancer cells, leading to cell death or inhibition of tumor growth.^4^, ^19^ The mechanism is primarily attributed to the ability of BL to stimulate the production of ROS by photoreceptors in mitochondria, thereby inducing cell apoptosis.^3^ Teng et al. (2023) observed that tumor cell proliferation inhibition and apoptosis were significantly influenced by BL‐induced ROS production, affecting mitochondrial membrane potential and the expression of mitochondrial apoptosis‐related genes (Bax, Bad, and Bcl‐2).^3^ This effect was observed at different times after exposure to BL, ranging from 6 to 72 h.^2^, ^3^, ^12^, ^16^, ^27^, ^29^, ^35^, ^37^ Sato et al. (2013) demonstrated an increase in superoxide anion after 6 and 12 h of irradiation and loss of mitochondrial membrane potential after irradiation with BL, mainly at longer irradiation times, while Nishio et al. (2022) observed this effect at 12 and 24 h. Takeuchi et al. (2023) noted that this effect extended for 48 h, and Patel et al. (2014) observed that it was more pronounced at 72 h with 45 J/cm^2^.^2^, ^12^, ^16^, ^27^ Additionally, some genes associated with endoplasmic reticulum stress, including *HSPA5*, *XBP1*, *EIF2AK3*, *DDIT3*, *NFE2L2*, and *GADD45a*, were significantly upregulated following exposure to 457 nm light. In contrast, only *XBP1* and *GADD45a* demonstrated significant upregulation after exposure to 475 nm light.^28^ Zhao et al. (2024) observed that a sharp decrease in *NOX5* gene expression directly affected ROS levels and impeded cell cycle progression, and the increased ROS levels significantly reduced the efficacy of BL.^41^ The modulation of ROS expression requires caution because exceeding a critical threshold of ROS production can negatively impact cell viability, leading to cytotoxic effects that may induce cell death and increase cell proliferation, which depends on the dose used and the frequency of radiation.^32^

Additionally, there was a reduction or loss of the membrane potential^3^, ^12^, ^16^, ^35^, ^36^, ^37^ after BL irradiation, in the production and consumption of ATP as well as a decrease in the rate of glycolysis and in the glycolytic reserve.^32^ On the other hand, two studies reported no change in ROS production.^26^, ^32^

##### Cell migration and invasion

The migratory and/or invasive capacity of tumor cells was verified by 12 studies. In general, the results indicated a significant suppression of the migratory and invasive capacity of cancer cells when exposed to BL irradiation, suggesting a strong anti‐metastatic potential of this therapy (Table 4).

The migratory and/or invasive capacity of tumor cells was verified by 12 studies.^2^, ^3^, ^13^, ^17^, ^19^, ^20^, ^22^, ^30^, ^33^, ^37^, ^38^, ^39^ Overall, the results indicated a significant suppression of the migratory and invasive capacity of cancer cells when exposed to BL irradiation, suggesting a strong anti‐metastatic potential of this therapy.^2^, ^3^, ^13^, ^19^, ^20^, ^30^, ^33^, ^37^, ^38^, ^39^ Only one study did not identify a decrease in the migration of breast cancer cells; however, it noted an inhibition in cellular invasion when cells were exposed to 482 J/cm^2^.^22^ In the study developed by Yoshimoto et al. (2024), cancer cells treated with conditioned medium from M2 macrophages submitted to BL irradiation showed decreased cellular migration.^17^

Molecular mechanisms associated with the inhibition of cell migration and invasion after PBM with BL include the reduction or inhibition of matrix metalloproteinases (MMP2 and MMP9) through p38 phosphorylation, differential regulation of the expression of proteins associated with cell adhesion, such as E‐cadherin and N‐cadherin, vimentin, twist, and snail, and inhibition of the *MPK/MEK/ERK* signaling pathway.^19^, ^20^, ^30^

#### In vivo

In general, in vivo studies have shown that BL inhibits tumor growth, as seen through tumor shrinkage,^2^, ^17^, ^27^, ^34^, ^40^, ^42^ decreased cell viability^6^ and cell proliferation,^27^ in addition to reduced formation of metastases^4^, ^20^, ^40^ and increased survival.^4^, ^6^, ^36^, ^42^

Studies have shown the influence of BL on the expression of proteins and genes, such as the reduction in the expression of AKT2^34^ and suppression of the genes *CCL7*, *HPSE*, *MMP10*, *MYC*, *SET*, *SRC*, *SYK*, and *TGFβ1*.^4^ Takeuchi et al. (2023) observed an increase in the expression of cleaved PARP and Caspase‐3, while Yoshimoto et al. (2024) demonstrated that BL was able to reduce the expression of PD‐L1 and observed that tumors treated with BL exhibited a smaller number of cells positive for the F4/80 and CD163 proteins compared to the control group.^2^, ^17^ The production of ROS was observed by three studies that showed both a reduction^27^ and an increase after BL exposure^40^ and after exposure to the combination of BL with vitamin C.^14^

### Quality assessment

QUIN tool was used to guide the assessment of the quality of the in vivo studies.^7^ All in vitro studies presented low risk of bias (Table S2). Although most studies presented adequate description of methodology and outcomes, 18 studies failed to provide all the parameters needed to guarantee the reproducibility of BL irradiation as total energy (Joules), method of irradiation, irradiated area, and equipment power^3^, ^4^, ^11^, ^12^, ^13^, ^14^, ^16^, ^17^, ^18^, ^19^, ^20^, ^26^, ^33^, ^35^, ^36^, ^39^, ^40^ (Table S2).

**FIGURE 3:**
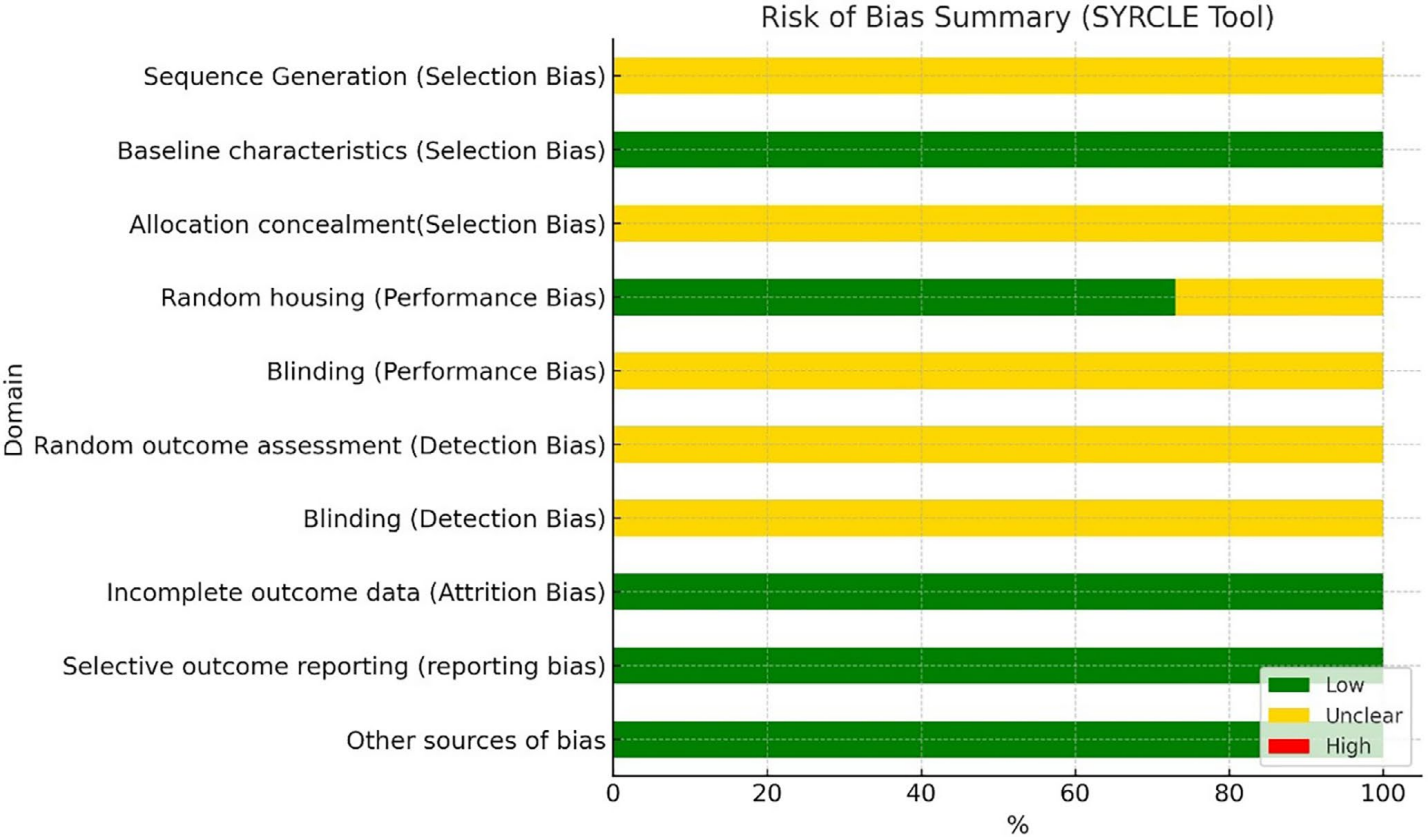
Risk of bias assessment of the in vivo studies according to SYRCLE tool per domain.

The risk of bias of the in vivo studies revealed that none of the authors clearly reported the sequence generation, allocation concealment, blinded allocation, randomization of outcome, and blinding of the outcome assessor (Figure 3 and Table S3). Only seven studies reported random housing. All included studies have comprehensive outcome‐based data and published intended results. Since many items evaluated by the SYRCLE^43^ were not explained in detail in the included studies, this leads to great difficulties and deviations in the interpretation of research bias. Nonetheless, the overall risk of bias was considered low to moderate. We have also observed a lack of uniformity in the description of dosimetric parameters of BL applied in the animal models.

## DISCUSSION

The available evidence in the literature regarding the biological effects of BL on various types of cancer was qualitatively described in this systematic review, which was based on 37 articles with preclinical models (in vitro and in vivo). Most studies used an in vitro approach alone, while 11 studies also evaluated the in vivo effects, and only one developed an exclusively in vivo methodology. The survey was conducted with studies published since 2002, 31 of which were published in the last 10 years, demonstrating a recent significant increase in interest and importance of understanding the effects of PBM with BL on cancer.

The studies evaluated adopted a broad methodological approach, both in vitro and in vivo, to investigate the effects of irradiation on different types of cancer. Methods that evaluated the viability, proliferation, and migration of tumor cells, effects on cell death, the expression of proteins and genes associated with neoplastic mechanisms, and the formation of reactive oxygen species were used to investigate the action of PBM with BL on the process of carcinogenesis. These findings reflect the search for a holistic understanding of the mechanisms underlying the effects of BL. The diversity of techniques used highlights the complexity of the analyses and the need for an integrated approach to capture the multifaceted effects of irradiation on tumors.

In vitro studies have revealed an important anti‐neoplastic effect of PBM with BL with direct effects on tumor cells, such as induction of programmed cell death, reduction of their viability and capacity for proliferation, migration, and invasion, evidencing a possible reduction in metastatic capacity.^2^, ^3^, ^4^, ^6^, ^10^, ^11^, ^12^, ^13^, ^15^, ^16^, ^17^, ^18^, ^19^, ^20^, ^21^, ^27^, ^28^, ^29^, ^30^, ^31^, ^32^, ^33^, ^34^, ^35^, ^36^, ^37^, ^38^, ^39^, ^41^ PBM with BL decreased cell viability in a dose‐dependent manner for the different types of tumor cells analyzed.^2^, ^3^, ^6^, ^10^, ^11^, ^12^, ^13^, ^15^, ^16^, ^19^, ^20^, ^27^, ^28^, ^29^, ^30^, ^31^, ^32^, ^33^, ^34^, ^36^, ^37^, ^38^, ^39^, ^41^

The evaluation of cell viability is essential in the studies that evaluate the efficacy of antineoplastic therapies. Across the studies, a time‐dependent response to BL irradiation was observed, with more pronounced effects typically occurring between 24 and 48 h post‐irradiation. These findings highlight the importance of optimizing exposure duration. However, variations in response kinetics among different cell types suggest that temporal factors must be carefully considered in future experimental designs. Some studies did not observe significant effects on cell viability with the isolated use of BL, requiring more comprehensive studies and repetition of protocols for a clearer analysis. It is also important to evaluate other possible influences, such as drug interactions and specific cell characteristics, to optimize its use in clinical contexts.

Studies on cell proliferation highlight the efficacy of PBM with BL, despite the use of different methodological approaches. The consistent observation of a reduction in the number and size of colonies, especially when combined with chemotherapy, suggests significant therapeutic potential.^3^, ^4^, ^10^, ^12^, ^16^, ^19^, ^28^, ^29^, ^30^, ^33^, ^34^, ^35^, ^37^, ^39^, ^41^ However, variations in cell cycle outcomes after BL irradiation raise questions about the exact mechanisms involved. While some studies indicate an increase in the G0/G1 phase and a reduction in G2/M and S phases, others do not report these changes consistently. These discrepancies may reflect differences in cell models, irradiation doses, or other experimental factors. Furthermore, changes in protein and gene expression suggest a complex interaction of BL with different cell signaling pathways, with implications for proliferation and apoptosis.^2^, ^3^, ^10^, ^13^, ^14^, ^15^, ^16^, ^18^, ^19^, ^21^, ^28^, ^29^, ^31^, ^32^, ^33^, ^34^, ^35^, ^36^, ^38^, ^39^ The precise understanding of these mechanisms requires further investigation, and the specificity of the effects observed in different cell types and the possibility of adverse effects also need to be addressed to fully assess the therapeutic potential of BL therapy.

PBM with BL was also effective in promoting tumor cell death, with an increase in apoptosis and in the expression of associated proteins, such as Caspase‐3 and PARP.^2^, ^16^, ^19^, ^21^, ^32^, ^34^, ^35^, ^36^ These findings suggest that BL can effectively induce programmed cell death, a vital mechanism in the regulation of cell growth and tissue homeostasis of malignant tumors.^2^, ^21^ In addition, BL was able to decrease the expression of proteins associated with autophagy, such as Bcl‐2,^3^, ^19^, ^34^, ^36^ bringing a possible elucidation of the molecular mechanisms associated with increased apoptosis. Still, the mechanisms are not fully elucidated; further studies are needed to validate and elucidate the mechanisms underlying the observed effects of BL on cell death.

The positive effects of BL irradiation extended to the process of cell migration and invasion, where a significant suppression of these processes was observed in almost all studies after irradiation with BL, suggesting a strong anti‐metastatic potential. Some studies also evaluated molecular mechanisms associated with this potential, such as reduction or inhibition of matrix metalloproteinases (MMP2 and MMP9) through p38 phosphorylation, differential regulation of the expression of proteins associated with cell adhesion, such as E‐cadherin and N‐cadherin, vimentin, twist, and snail, and inhibition of the MPK/MEK/ERK signaling pathway.^19^, ^20^, ^30^

In addition, some studies have described other effects of PBM with BL on tumor cells. The study by Yoshimoto et al. (2024) demonstrated that irradiation with BL can modify the tumor microenvironment by reducing the polarization of tumor‐associated macrophages (TAMs) and the secretion of VEGF, in addition to decreasing the expression of PD‐L1 in cancer cells. These effects suggest that BL can weaken the support for tumor growth and potentially increase the efficacy of immune therapies, offering a promising new approach for the treatment of cancer.^17^

Analysis of the effects of BL in in vivo studies demonstrated evident anti‐neoplastic effects. Studies have consistently demonstrated inhibition of tumor growth in mice and distant metastases, also noted through the decreased expression of genes associated with the metastasis process, such as *CCL7*, *HPSE*, *MMP10*, *MYC*, *SET*, *SRC*, *SYK*, and *TGFβ1*. Most importantly, despite the differences in the dosimetric parameters used for BL irradiation in vivo, the studies indicated improved survival. Furthermore, Yang et al. (2024) showed that BL irradiation increased intracellular ROS and promoted the activation of innate immunity, which was able to decrease subcutaneous tumor volume and pulmonary metastatic tumor nodules through activated macrophages and neutrophils.^40^ However, these promising results must be interpreted with caution as many studies lack specific methodological details, including radiant exposure, total energy, equipment specifications, and exposure time, among others, that may compromise the replicability and interpretation of results. This gap in literature highlights the need for more comprehensive and detailed research to fully understand the mechanisms underlying the in vivo anti‐tumor effects of PBM with BL.

The reviewed studies employed a broad range of dosimetric parameters and irradiation devices. While wavelengths between 450 and 470 nm were most common, some studies extended this range considerably. Findings from Jiang et al. (2024) demonstrated that higher irradiances (e.g., 50 mW/cm^2^) induced greater oxidative stress and mitochondrial dysfunction, whereas lower irradiances showed minimal biological effects. These results reinforce the need for precise dosimetric calibration to elicit anti‐tumor effects of BL while minimizing off‐target effects.^29^

The quality assessment of the in vitro studies included in this systematic review showed a low risk of bias. However, in vivo studies presented more biases than in vitro studies. These aspects are inherently more challenging to implement in studies involving blue light irradiation, as the visible nature of the light makes blinding of both the intervention and the outcome assessor more difficult. Moreover, methodological details regarding BL irradiation were not consistently reported across the in vivo studies. In the context of PBM, accurate reporting of dosimetric parameters is particularly critical to ensure study quality and reproducibility. While some studies provided a wide range of information, including irradiance, exposure time, radiation exposure, number of irradiated points, and frequency of sessions, others do not mention sufficient data for experimental replication (e.g., equipment power, total energy, and irradiated area). The lack of this information may impair the reproduction of experiments and comparison between the studies, indicating a potential limitation regarding the in vitro and in vivo experimental protocols available in the literature.

It is important to emphasize that while BL (400–500 nm) has emerged as a promising approach for cancer therapy capable of selectively inducing cytotoxic effects in tumor cells through the activation of endogenous chromophores and subsequent reactive oxygen species (ROS) generation – its combination with photosensitizers in photodynamic therapy (PDT) has demonstrated even greater anti‐tumor efficacy in preclinical models, suggesting a potential dual therapeutic role for BL.^11^, ^26^ In contrast, PBM using red and near‐infrared (NIR) wavelengths commonly applied to manage the side effects of cancer treatments due to their well‐documented benefits in tissue repair and regeneration, inflammation modulation, and pain relief was historically associated with possible tumor‐promoting effects (44). These effects are thought to occur primarily through the stimulation of mitochondrial activity and enhanced cellular proliferation, which, in certain contexts, may inadvertently support tumor progression.^44^ Together, these findings underscore the critical importance of carefully selecting both the wavelength and treatment parameters to ensure safe and effective use of light‐based therapies in oncology.

Despite the therapeutic potential of BL as an anti‐cancer therapy, its suitability may vary significantly across different cancer types, largely influenced by tumor location, tissue optical properties, and cellular sensitivity. Superficial skin tumors and early‐stage head and neck cancer patients may benefit most from blue BL therapy because of the limited tissue penetration depth (~1–3 mm) associated with blue wavelengths.^8^, ^15^ In contrast, deeply seated tumors pose a significant challenge due to insufficient light delivery, reducing therapeutic efficacy. In addition, in pigmented tumors such as melanoma, melanin strongly absorbs BL, and its effects may be impaired.^13^ Furthermore, the heterogeneity of cancer cells, especially related to their antioxidant defenses, can lead to differential responses to BL, indicating that irradiation parameters may need to be specifically optimized for each cancer subtype.

Practical challenges to the clinical translation of BL therapy include the absence of standardized protocols for irradiation in both in vitro and in vivo studies, which limits the ability to compare outcomes and establish effective treatment guidelines. Thus, careful optimization of treatment parameters is essential for maximizing BL efficacy. In addition, ensuring consistent light distribution within tumors, minimizing damage to adjacent healthy tissues, and overcoming the limited penetration depth of BL remain major technical obstacles. Advances in light delivery technologies, including fiber optics and nanoparticles, as well as the integration of BL therapy into multimodal treatment strategies, may help address these issues and improve clinical applicability.

This review offers a broad and up‐to‐date overview of the use of PBM with blue light in oncology, based on studies published over the past 22 years. Some limitations should be considered. Our search included only a few languages, which may have led to the exclusion of studies published in other languages, such as Chinese and Russian. We were also not able to contact the authors of all included studies to clarify missing methodological details. Additionally, we focused on peer‐reviewed publications, which may have limited the inclusion of preprint studies or those with negative results. Even so, our approach prioritized the inclusion of high‐quality evidence and aimed to ensure consistency and reliability in the data analyzed.

This systematic review highlights the promising antitumor effects of PBM with blue light (BL), demonstrated across a range of in vitro and in vivo models. These effects include reduced tumor cell viability and proliferation, induction of apoptosis, inhibition of migration and invasion, and modulation of oxidative stress and gene expression. Most importantly, in vivo models also showed decreased tumor growth and metastasis, with activation of immune responses and improved overall survival. However, for clinical translation of these findings, future studies should prioritize in vitro 3D models that more closely represent the tumor microenvironment and, importantly, focus on the standardization of BL irradiation parameters. Establishing consistent protocols, including wavelength, fluence, exposure time, and application mode, will be essential to improve reproducibility and enhance the translational potential of BL in oncology.

## AUTHOR CONTRIBUTIONS

BESL performed the literature search, data extraction, conducted the formal analysis and interpretation; RBN performed the literature search, data extraction, conducted the formal analysis and interpretation, drafted and edited the manuscript; APM conducted the formal analysis and interpretation; MSM conducted data collection and critically reviewed the manuscript; RBC conducted the formal analysis and interpretation, critically reviewed and edited the manuscript; RML contributed the formal analysis and interpretation, critically reviewed and edited the manuscript; and MFSDR contributed to data extraction, conducted the formal analysis and interpretation, drafted the manuscript, critically reviewed and edited the manuscript, and supervised the project.

## FUNDING INFORMATION

This study was supported by São Paulo Research Foundation (FAPESP, grant number 2018/08540‐8), National Council for Scientific and Technological Development (CNPq, grant numbers 312269/2022‐6 and 403067/2024‐3) and Coordenação de Aperfeiçoamento de Pessoal de Nível Superior – Brasil (CAPES, Bárbara Evelyn Santos de Lima process 88887.818196/2023‐00, Rebeca Barros Nascimento process 88887.007733/2024‐00 and Ana Paula Mariano process 88887.085032/2024‐00).

## CONFLICT OF INTEREST STATEMENT

The authors declare that they have no conflict of interest.

## Supporting information


Data S1.

## Data Availability

Research data are not shared.

## APPENDIX A Terms used in research

(Cancer[Title/Abstract]) OR (Malignancy[Title/Abstract]) OR (Neoplasms[Title/Abstract]) OR (Malignant Neoplasm[Title/Abstract]) OR (MalignantNeoplasms[Title/Abstract]) OR (Neoplasia[Title/Abstract]) OR (Tumor[Title/Abstract]) OR (Tumors[Title/Abstract]) OR (cancer cell[Title/Abstract]) OR (neoplasm cell[Title/Abstract]) OR (neoplastic cell[Title/Abstract]) AND (blue light[Title/Abstract]) OR (blue photobiomodulation[Title/Abstract]) OR (blue light therapy[Title/Abstract]) OR (blue light irradiation[Title/Abstract]) OR (blue laser[Title/Abstract]) OR (blue light[Title/Abstract]).
