# Supplementary material for: Harnessing blue light photobiomodulation for cancer therapy: Evidence from a systematic review
Source: Photochem Photobiol. 2025 Aug 28;102(2):370–99. doi: 10.1111/php.70025 (PMC13005293; doi:10.1111/php.70025)
Supplement: Supplementary file 1 — Data S1. [file PHP-102-370-s001.docx]

## Harnessing Blue Light Photobiomodulation for Cancer Therapy: Evidence from a Systematic Review

Bárbara Evelyn Santos de Lima MSc^1^, Rebeca Barros Nascimento PhD^1^, Ana Paula Mariano Santos Ginez^1^, Maria Stella Moreira PhD^2,3^ Rebeca Boltes Cecatto PhD^1^, Rodrigo Labat Marcos PhD^1^, Maria Fernanda Setúbal Destro Rodrigues PhD^1^

^1^Medicine-Biophotonics PostGraduate Program, Universidade Nove de Julho/UNINOVE, São Paulo, Brazil

^2^Department of Stomatology, School of Dentistry, University of São Paulo, São Paulo, Brazil

^3^Department of Oral Medicine, A.C. Camargo Cancer Center, São Paulo, Brazil

Corresponding Author:

Maria Fernanda Setúbal Destro Rodrigues, PhD, Post Graduate Program in Medicine-Biophotonics, Universidade Nove de Julho, UNINOVE Rua Vergueiro 235/249, Liberdade, CEP 01504-001 São Paulo, Brasil. [fernandarodrigues@uni9.pro.br](mailto:fernandarodrigues@uni9.pro.br); fernandadestrorodrigues@gmail.com

**Supplementary table 1:** General data of the included studies**.**

| **Author** | **Year** | **Country** | **Study type** | **Cancer type** |
| --- | --- | --- | --- | --- |
| Ohara et al. (10) | 2002 | Japan | *In vitro* | Melanoma |
| Ohara et al. (6) | 2002 | Japan | *In vitro* and *In vivo* | Leukemia |
| Ohara et al. (42) | 2003 | Japan | *In vivo* | Skin cancer |
| Lockwood et al. (26) | 2005 | Taiwan | *In vitro* | Squamous cells carcinoma of the gum |
| Sparsa et al. (11) | 2010 | France | *In vitro* | Melanoma |
| Sato et al. (12) | 2013 | Japan | *In vitro* | Melanoma |
| Patel et al. (27) | 2014 | USA | *In vitro* and *In vivo* | Oral squamous cell carcinoma |
| Matsumoto et al. (21) | 2014 | Japan | *In vitro* | Colon cancer |
| Oh et al. (36) | 2016 | Korea | *In vitro* and *In vivo* | Lymphoma |
| Oh et al. (20) | 2017 | Korea | *In vitro* and *In vivo* | Colon cancer and fibrosarcoma |
| Yan et al. (19) | 2018 | China | *In vitro* | Colon adenocarcinoma |
| Yoshimoto et al. (18) | 2018 | Japan | *In vitro* | Colon cancer |
| Kim et al. (34) | 2020 | Korea | *In vitro* and *In vivo* | Pancreatic cancer |
| Xia et al. (30) | 2021 | China | *In vitro* | Bladder and urothelial cancer |
| He et al. (39) | 2021 | China | *In vitro* | Osteosarcoma |
| Zhuang et al. (35) | 2022 | China | *In vitro* | Leukemia |
| Hegmann et al. (32) | 2022 | Germany | *In vitro* | Bladder cancer |
| Nishio et al. (16) | 2022 | Japan | *In vitro* | Melanoma |
| Takeuchi et al. (2) | 2023 | Japan | *In vitro* and *In vivo* | Synovial sarcoma |
| Zhou et al. (13) | 2023 | Japan | *In vitro* | Melanoma |
| Yang et al. (14) | 2023 | China | *In vitro* and *In vivo* | Melanoma |
| Jiang et al. (37) | 2023 | China | *In vitro* | Lung cancer |
| Teng et al. (3) | 2023 | China | *In vitro* | Hepatic cancer |
| Silva et al. (22) | 2023 | Brazil | *In vitro* | Breast cancer |
| Oh et al. (4) | 2023 | Korea | *In vitro* and *In vivo* | lung cancer, colorectal câncer, hepatic câncer, pancreatic câncer, Burkitt's lymphoma |
| Wang et al.(33) | 2023 | China | *In vitro* | Carcinoma hepatocelular |
| Farias et al. (24) | 2024 | Brazil | *In vitro* | Breast cancer |
| Sturm et al. (31) | 2024 | Germany | *In vitro* | Bladder cancer |
| Yoshimoto et al. (17) | 2024 | Japan | *In vitro and In vivo* | Colon cancer |
| Yang et al. (40) | 2024 | China | *In vitro and in vivo* | Skin cancer |
| Jiang et al. (29) | 2024 | China | *In vitro* | Oral squamous cell carcinoma |
| Qin et al. (15) | 2024 | China | *In vitro* | Melanoma |
| Yang et al. (38) | 2024 | China | *In vitro* | Osteosarcoma |
| Jiang et al. (28) | 2024 | China | *In vitro* | Oral squamous cell carcinoma |
| Ibrahim et al. (25) | 2024 | Malaysis | *In vitro* | Breast cancer |
| Farias et al. (23) | 2024 | Brazil | *In vitro* | Breast cancer |
| Zhao et al. (41) | 2024 | China | *In vitro* | Papillary thyroid carcinoma |
|  |  |  |  |  |
|  |  |  |  |  |
|  |  |  |  |  |

**Suplemmentary table 2:** Evaluation of the risk of bias of the *in vitro* studies according with the QUIN tool.

| **Author** | **Q1** | **Q2** | **Q3** | **Q4** | **Q5** | **Q6** | **Q7** | **Q8** | **Q9** | **Q10** | **Q11** | **Q12** | **Final score (%)** | **Risk of bias** |
| --- | --- | --- | --- | --- | --- | --- | --- | --- | --- | --- | --- | --- | --- | --- |
| Ohara et al. (10) | 2 | NA | NA | 2 | 2 | NA | NA | 2 | NA | NA | 2 | 2 | 100 | Low risk |
| Ohara et al. (6) | 2 | NA | NA | 2 | 2 | NA | NA | 2 | NA | NA | 2 | 2 | 100 | Low risk |
| Lockwood et al. (26) | 2 | NA | NA | 2 | 1 | NA | NA | 2 | NA | NA | 2 | 2 | 91.66 | Low risk |
| Sparsa et al. (11) | 2 | NA | NA | 2 | 1 | NA | NA | 2 | NA | NA | 2 | 2 | 91.66 | Low risk |
| Sato et al. (12) | 2 | NA | NA | 2 | 1 | NA | NA | 2 | NA | NA | 2 | 2 | 91.66 | Low risk |
| Patel et al. (27) | 2 | NA | NA | 2 | 2 | NA | NA | 2 | NA | NA | 2 | 2 | 100 | Low risk |
| Matsumoto et al. (21) | 2 | NA | NA | 2 | 2 | NA | NA | 2 | NA | NA | 2 | 2 | 100 | Low risk |
| Oh et al. (36) | 2 | NA | NA | 2 | 1 | NA | NA | 2 | NA | NA | 2 | 2 | 91.66 | Low risk |
| Oh et al. (20) | 2 | NA | NA | 2 | 1 | NA | NA | 2 | NA | NA | 2 | 2 | 91.66 | Low risk |
| Yan et al. (19) | 2 | NA | NA | 2 | 1 | NA | NA | 2 | NA | NA | 2 | 2 | 91.66 | Low risk |
| Yoshimoto et al. (18) | 2 | NA | NA | 2 | 1 | NA | NA | 2 | NA | NA | 2 | 2 | 91.66 | Low risk |
| Kim et al. (34) | 2 | NA | NA | 2 | 2 | NA | NA | 2 | NA | NA | 2 | 2 | 100 | Low risk |
| Xia et al. (30) | 2 | NA | NA | 2 | 2 | NA | NA | 2 | NA | NA | 2 | 2 | 100 | Low risk |
| He et al. (39) | 2 | NA | NA | 2 | 1 | NA | NA | 2 | NA | NA | 2 | 2 | 91.66 | Low risk |
| Zhuang et al. (35) | 2 | NA | NA | 2 | 1 | NA | NA | 2 | NA | NA | 2 | 2 | 91.66 | Low risk |
| Hegmann et al. (32) | 2 | NA | NA | 2 | 2 | NA | NA | 2 | NA | NA | 2 | 2 | 100 | Low risk |
| Nishio et al. (16) | 2 | NA | NA | 2 | 1 | NA | NA | 2 | NA | NA | 2 | 2 | 91.66 | Low risk |
| Takeuchi et al. (2) | 2 | NA | NA | 2 | 1 | NA | NA | 2 | NA | NA | 2 | 2 | 100 | Low risk |
| Zhou et al. (13) | 2 | NA | NA | 2 | 1 | NA | NA | 2 | NA | NA | 2 | 2 | 100 | Low risk |
| Yang et al. (14) | 2 | NA | NA | 2 | 1 | NA | NA | 2 | NA | NA | 2 | 2 | 100 | Low risk |
| Jiang et al. (37) | 2 | NA | NA | 2 | 2 | NA | NA | 2 | NA | NA | 2 | 2 | 100 | Low risk |
| Teng et al. (3) | 2 | NA | NA | 2 | 1 | NA | NA | 2 | NA | NA | 2 | 2 | 100 | Low risk |
| Silva et al. (22) | 2 | NA | NA | 2 | 2 | NA | NA | 2 | NA | NA | 2 | 2 | 100 | Low risk |
| Oh et al. (4) | 2 | NA | NA | 2 | 1 | NA | NA | 2 | NA | NA | 2 | 2 | 91.66 | Low risk |
| Wang et al.(33) | 2 | NA | NA | 2 | 1 | NA | NA | 2 | NA | NA | 2 | 2 | 91.66 | Low risk |
| Farias et al. (24) | 2 | NA | NA | 2 | 2 | NA | NA | 2 | NA | NA | 2 | 2 | 100 | Low risk |
| Sturm et al. (31) | 2 | NA | NA | 2 | 2 | NA | NA | 2 | NA | NA | 2 | 2 | 100 | Low risk |
| Yoshimoto et al. (17) | 2 | NA | NA | 2 | 1 | NA | NA | 2 | NA | NA | 2 | 2 | 91.66 | Low risk |
| Yang et al. (40) | 2 | NA | NA | 2 | 1 | NA | NA | 2 | NA | NA | 2 | 2 | 91.66 | Low risk |
| Jiang et al. (29) | 2 | NA | NA | 2 | 2 | NA | NA | 2 | NA | NA | 2 | 2 | 100 | Low risk |
| Qin et al. (15) | 2 | NA | NA | 2 | 2 | NA | NA | 2 | NA | NA | 2 | 2 | 100 | Low risk |
| Yang et al. (38) | 2 | NA | NA | 2 | 2 | NA | NA | 2 | NA | NA | 2 | 2 | 100 | Low risk |
| Jiang et al. (28) | 2 | NA | NA | 2 | 2 | NA | NA | 2 | NA | NA | 2 | 2 | 100 | Low risk |
| Ibrahim et al. (25) | 2 | NA | NA | 2 | 2 | NA | NA | 2 | NA | NA | 2 | 2 | 100 | Low risk |
| Farias et al. (23) | 2 | NA | NA | 2 | 2 | NA | NA | 2 | NA | NA | 2 | 2 | 100 | Low risk |
| Zhao et al. (41) | 2 | NA | NA | 2 | 2 | NA | NA | 2 | NA | NA | 2 | 2 | 100 | Low risk |

Adequately Specified (Score=2), Inadequately Specified (Score=1), Not Specified (Score=0) and NA=Not Applicable. The final score was calculated using the formula: (Sum of the scores×100)/2 × number of applicable criteria. The study was classified as high risk of bias if the final score was >70%, medium risk if the final score was 50-70% and low risk of bias if the final score was <50%.

QUIN’s questions for review author’s judgment:

Q1 - Clearly stated aims/ objectives

Q2 - Detailed explanation of sample size calculation

Q3 - Detailed explanation of sampling technique

Q4 – Details of comparison group

Q5 - Detailed explanation of methodology

Q6 - Operator details

Q7 - Randomization

Q8 – Method of measurement of outcome

Q9 - Outcome assessor details

Q10 - Blinding

Q11 - Statistical analysis

Q12 - Presentation of results

**Suplemmentary table 3**: Evaluation of the risk of bias of the *in vivo* studies according with the SYRCLE’s risk of bias tool for animal studies.

| **Author** | Sequence generation | Baseline characteristics | Allocation concealment | Random housing | Blinding (Performance Bias) | Random outcome assessment | Blinding (Detection Bias) | Incomplete outcome data | Selective outcome reporting | Other sources of bias |
| --- | --- | --- | --- | --- | --- | --- | --- | --- | --- | --- |
|  | **Q1** | **Q2** | **Q3** | **Q4** | **Q5** | **Q6** | **Q7** | **Q8** | **Q9** | **Q10** |
| Ohara et al. (2002) (6) |  |  |  |  |  |  |  |  |  |  |
| Ohara et al. (2003) (42) |  |  |  |  |  |  |  |  |  |  |
| Patel et al. (2014) (27) |  |  |  |  |  |  |  |  |  |  |
| Oh et al. (2016) (36) |  |  |  |  |  |  |  |  |  |  |
| Oh et al. (2017) (20) |  |  |  |  |  |  |  |  |  |  |
| Kim et al. (2020) (34) |  |  |  |  |  |  |  |  |  |  |
| Takeuchi et al. (2023) (2) |  |  |  |  |  |  |  |  |  |  |
| Yang et al. (2023) (14) |  |  |  |  |  |  |  |  |  |  |
| Oh et al. (2023) (4) |  |  |  |  |  |  |  |  |  |  |
| Yoshimoto et al. (2024) (17) |  |  |  |  |  |  |  |  |  |  |
| Yang et al. (2024) (40) |  |  |  |  |  |  |  |  |  |  |

Yes=green, Unclear=yellow, No=red

**Syrcle’s questions for review author’s judgment:**

Q1 - Was the allocation sequence adequately generated and applied?

Q2 - Were the groups similar at baseline or were they adjusted for confounders in the analysis?

Q3 - Was the allocation adequately concealed?

Q4 - Were the animals randomly housed during the experiment?

Q5 - Were the caregivers and/or investigators blinded from knowledge which intervention each animal received during the experiment?

Q6 - Were animals selected at random for outcome assessment?

Q7 - Was the outcome assessor blinded?

Q8 - Were incomplete outcome data adequately addressed?

Q9 - Are reports of the study free of selective outcome reporting?

Q10 - Was the study apparently free of other problems that could result in high risk of bias?
